# Regulatory role of endoplasmic reticulum resident chaperone protein ERp29 in anti-murine β-coronavirus host cell response

**DOI:** 10.1016/j.jbc.2022.102836

**Published:** 2022-12-23

**Authors:** Abhishek Bose, Grishma Kasle, Rishika Jana, Mahua Maulik, Deepthi Thomas, Vaishali Mulchandani, Priyanka Mukherjee, Michael Koval, Jayasri Das Sarma

**Affiliations:** 1 Department of Biological Sciences, Indian Institute of Science Education and Research Kolkata, Mohanpur, India; 2 Departments of Medicine and Cell Biology, Emory University School of Medicine, Atlanta, Georgia, USA

**Keywords:** β-coronavirus infection, murine coronavirus (mCoV), MHV-A59, Connexin43, gap junctional intercellular communication (GJIC), ER stress, ERp29, astrocytes, ATF6, activating transcription factor 6, BiP, binding immunoglobulin protein, β-CoV, β-coronavirus DAPI, 4, 6-diamidino-2-phenylindole, DBT, delayed brain tumor, DMEM, Dulbecco's modified Eagle's medium, ER, endoplasmic reticulum, eIF2α, eukaryotic translation initiation factor-2α, ER-ERGIC, ER-Golgi intermediate complex, FBS, fetal bovine serum, GJIC, gap junctional intercellular communication, HBSS, Hank's Balanced Salt Solution, IRE1, inositol-requiring protein 1, LY, Lucifer yellow, mCoV, murine β-CoV, MHV-A59, mouse hepatitis virus strain A59, MOI, multiplicities of infection, 4-PBA, 4-phenylbutyrate, PERK, PKR-like ER kinase, p.i., postinfection, UPR, unfolded protein response

## Abstract

Gap junctional intercellular communication (GJIC) involving astrocytes is important for proper CNS homeostasis. As determined in our previous studies, trafficking of the predominant astrocyte GJ protein, Connexin43 (Cx43), is disrupted in response to infection with a neurotropic murine β-coronavirus (MHV-A59). However, how host factors are involved in Cx43 trafficking and the infection response is not clear. Here, we show that Cx43 retention due to MHV-A59 infection was associated with increased ER stress and reduced expression of chaperone protein ERp29. Treatment of MHV-A59–infected astrocytes with the chemical chaperone 4-sodium phenylbutyrate increased ERp29 expression, rescued Cx43 transport to the cell surface, increased GJIC, and reduced ER stress. We obtained similar results using an astrocytoma cell line (delayed brain tumor) upon MHV-A59 infection. Critically, delayed brain tumor cells transfected to express exogenous ERp29 were less susceptible to MHV-A59 infection and showed increased Cx43-mediated GJIC. Treatment with Cx43 mimetic peptides inhibited GJIC and increased viral susceptibility, demonstrating a role for intercellular communication in reducing MHV-A59 infectivity. Taken together, these results support a therapeutically targetable ERp29-dependent mechanism where β-coronavirus infectivity is modulated by reducing ER stress and rescuing Cx43 trafficking and function.

Coronaviruses are a group of enveloped viruses with single-stranded, non-segmented, and positive-sense RNA genomes (1, 2). β-coronaviruses (β-CoVs) include five that infect human hosts and cause respiratory diseases, such as COVID-19, as well as those that infect economically important vertebrates (*e.g.*, pig, chicken) (1). To date, most antiviral therapies directed toward β-CoVs target the virus in the form of neutralizing antibodies or mimetic peptides that bind to the spike protein, which have varying degrees of efficacy. By contrast, therapeutic approaches that target host factors have had varying degrees of efficacy. Understanding host responses to β-CoV infection has the potential to identify novel targets with therapeutic potential.

The endoplasmic reticulum (ER) is the most commonly hijacked "niche" during viral infection (3). It is well characterized that β-CoV infection induces ER stress and activates the unfolded protein response (UPR) in infected cells (4). During acute infection, CoVs overburden the ER by producing large amounts of viral structural proteins that create double-membrane vesicles for the assembly of replication/transcription complexes required for viral budding (5). Specifically, the β-CoV viral spike (S) protein, that has the capacity to induce host cell-to-cell fusion, induces ER stress that stimulates the UPR, resulting in activation of the innate immune response (6, 7). Importantly, virus-induced ER stress can affect multiple host cellular pathways leading to cytopathic effects early in the disease process and thus can be a vital checkpoint to control CoV infection and minimize cellular damage.

ER stress is primarily sensed by the key sensor binding immunoglobulin protein, BiP, also known as glucose-regulated protein 78 (8). BiP is also one of the most abundant proteins in the ER, primarily binding nascent polypeptides to prevent their aggregation (9, 10). ER stress-induced upregulation of BiP triggers three different UPR signal transduction pathways involving transmembrane ER-resident proteins: inositol-requiring protein 1 (IRE1) (11), PKR-like ER kinase (PERK) (12), and activating transcription factor (ATF6) (13). Through these UPR pathways, the cell attempts to re-establish cellular homeostasis by moderating ER stress or, in the case of prolonged ER stress, promotes cellular death by activating apoptosis. Recognition of misfolded proteins may also occur independently of BiP by directly binding the luminal domain of IRE1, which causes IRE1 oligomerization and signaling, leading to a cytoprotective effect that is conserved across eukaryotes (14, 15). Specifically, IRE1 interacts with X-box-binding protein 1 (Xbp-1) mRNA to form a splice variant (Xbp-1(s)) through its endoribonuclease function. This spliced RNA encodes for a highly active transcription factor whose target genes encode cytoprotective proteins, reducing ER stress (16). The PERK pathway is responsible for the effects of UPR on translation. Activation of PKR-like ER Kinase is response to UPR causes phosphorylation of eukaryotic translation initiation factor-2α (eIF2α), a component of the EIF2 complex. Phosphorylated eIF2α inhibits ribosomes leading to a brief attenuation of baseline protein translation. The ATF6 pathway mediates a response that promotes transcription of genes-associated protein folding and ER-associated degradation pathway. Upon accumulation of misfolded proteins, ATF6 undergoes a regulated intramembrane proteolysis to release its cytosolic basic leucine zipper (bZip) transcription factor which migrates to the nucleus and mediated activation of UPR targeted genes (17).

Interaction of UPR proteins with chaperones and other cellular proteins has distinct implications in the pathology of several virus infections (18, 19). ER stress can also be induced chemically using pharmaceutical ER stress inducers such as tunicamycin, thapsigargin, and Brefeldin A (20) and is also associated with disease-associated mutations that induce protein misfolding (21, 22). Several agents have been shown to be effective at relieving ER stress and the UPR, underscoring the utility of pharmacologically targeting the UPR (23).

ER stress also is associated with changes in expression of several chaperones, including an ER resident thioredoxin family protein, ERp29 (24). ERp29 is expressed ubiquitously in almost all tissues, activated in response to ER stress, highly conserved across mammals, and has been extensively studied (25, 26, 27). The role of ERp29 in regulating non-enveloped polyomavirus infection has also been identified in earlier studies (28, 29). Specifically, ERp29 and other PDI family members were shown to facilitate the unfolding and ER retro-translocation of the polyomavirus VP1 protein, a key step in the virus infection cycle (29). However, its relation with CoV infection remains elusive.

Previous studies demonstrated that ERp29 has a key role in regulating the trafficking of a gap junction protein Connexin43 (Cx43). Downregulation of ERp29 leads to destabilized monomeric Cx43 oligomerization in the ER and causes intracellular accumulation of Cx43, thereby reducing trafficking to the plasma membrane and decreasing gap junction intercellular communication (GJIC) (30).

We have demonstrated that infection with neurotropic mouse hepatitis virus strain A59 (MHV-A59), a prototypic experimental murine β-CoV (mCoV), profusely infects astrocytes in the mouse brain as well as primary mixed glial cell cultures enriched in astrocytes. MHV-A59 infection of primary astrocytes also resulted in retention of Cx43 in the ER and ER-Golgi intermediate complex (ER-ERGIC) as opposed to non-infected cells that showed punctate Cx43 staining at the cell surface (31). From a mechanistic standpoint, it was further delineated that the virus hijacks the microtubule conduit which, under normal conditions, is used by Cx43 for trafficking to the cell surface (32). Whether mCoV-induced ER stress plays a role in the intracellular retention of Cx43 in the ER-ERGIC has not been addressed; however, this seemed to be a likely hypothesis, based on previous results demonstrating the control of Cx43 trafficking by the chaperone ERp29.

The current study was designed to understand the systemic interaction of host factors during infection to determine whether the ERp29/Cx43 axis has the capacity to be targeted as a therapeutic approach to inhibit viral replication and its associated cellular dystrophy. We found that MHV-A59 infection-induced ER stress, as indicated by increased BiP, increased Xbp splicing and reduced ERp29. Consistent with our previous studies, MHV-A59 infected cells retained Cx43 in the intracellular compartment. Inhibition of ER stress with the chemical chaperone 4-phenylbutyrate (4-PBA) increased ERp29 protein and rescued the trafficking of Cx43 to the cell surface in infected mouse primary astrocytes.

The mouse astrocytoma delayed brain tumor (DBT) cell line, which has been widely used in the MHV field to study the replication and propagation of different strains of the virus, was chosen as an immortalized cell line to further examine the effects of astrocyte MHV-A59 infection on ERp29 and Cx43. We produced a stably transfected DBT cell line expressing increased ERp29 (DBT-ERp29) and found that it was less susceptible to viral infectivity and syncytia formation. This reduced viral infectivity was associated with ERp29-mediated rescue of Cx43 trafficking to the cell surface and formation of functional gap junction channels, since Cx43 inhibitors reversed the ability of DBT-ERp29 cells to resist infection. These data suggest that ERp29 and Cx43 are potential therapeutic targets with the capacity to control β-coronaviral infectivity.

## Results

### MHV-A59 infected mouse primary astrocytes have decreased ERp29 expression associated with increased ER stress

**Figure 1:**
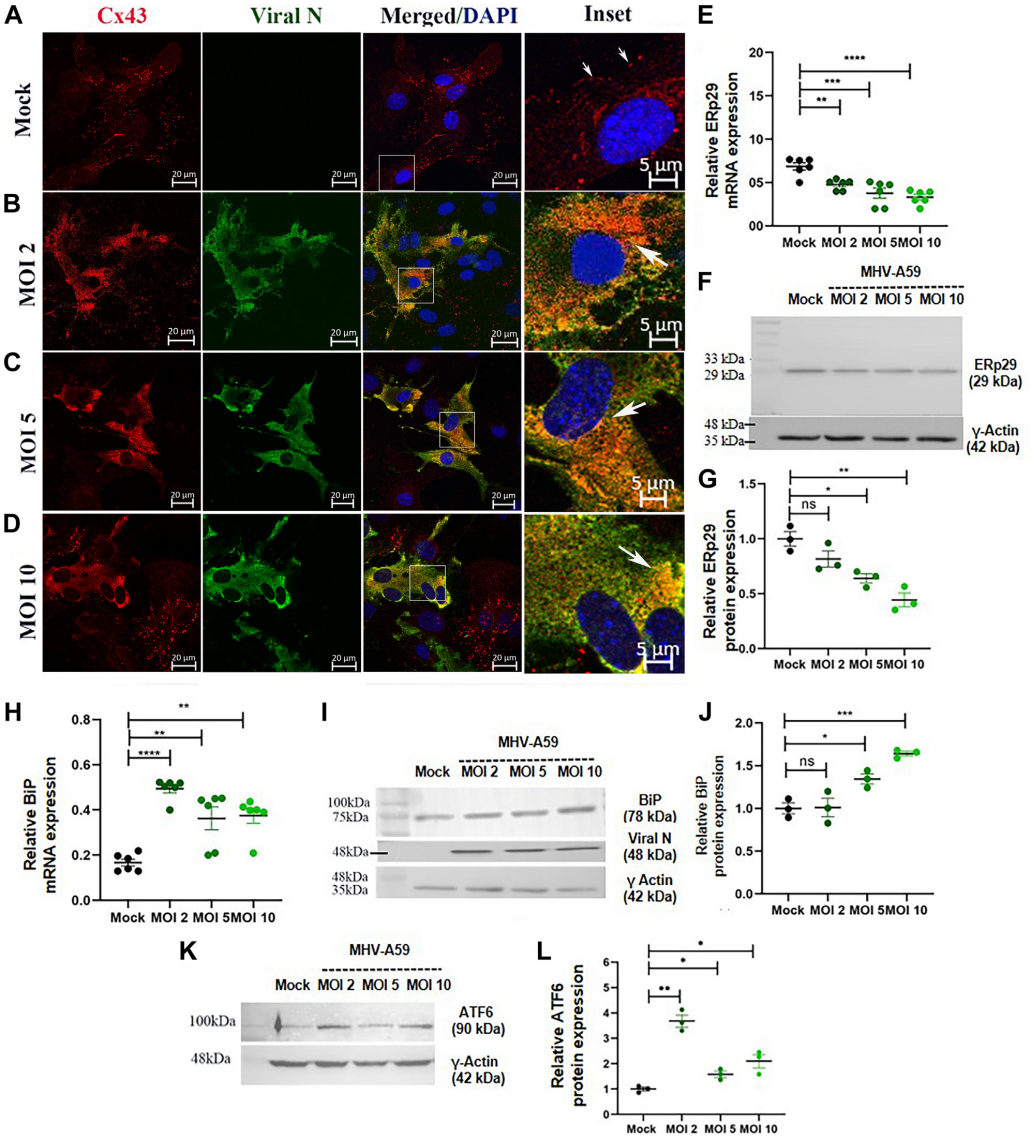
**Retention of Cx43, decrease in ERp29 and increase in BiP, ATF6 in response to MHV-A59 infection by primary astrocytes**. *A*–*D*, localization of Cx43 in mock (*A*) and MHV-A59 infected primary mouse astrocytes at MOI 2 (*B*), 5 (*C*), and 10 (*D*), 24 h p.i. analyzed by double immunolabeling followed by confocal immunofluorescence microscopy with anti-Cx43 (*red*) and antinucleocapsid (N) antibodies (*green*). *Small Arrows* and *large arrows* in insets show punctate Cx43 labeling at the cell–cell interfaces (A) or intracellular Cx43 retention in MHV-A59–infected astrocytes (*B–D*). *E*, scatter plot of ERp29 mRNA in mock or MHV-A59–infected astrocytes measured by qPCR. *F* and *G*, representative immunoblot and corresponding scatter plot of ERp29 protein in mock or MHV-A59–infected astrocytes. *H*, scatter plot of BiP mRNA in mock or MHV-A59 infected astrocytes measured by qPCR. *I* and *J*, representative immunoblot and corresponding scatter plot of BiP protein in mock or MHV-A59–infected astrocytes. *K* and *L*, representative immunoblot and corresponding scatter plot of ATF6 protein in mock and MHV-A59–infected astrocytes. γ-Actin was used as a loading control. Values are mean ± SEM analyzed by unpaired Student’s *t* test or one-way ANOVA (∗*p* < 0.05, ∗∗*p* < 0.01, ∗∗∗*p* < 0.001, ∗∗∗∗*p* < 0.0001, ns – not significant, N = 3). ATF6, activating transcription factor 6; BiP, binding immunoglobulin protein; MHV-A59, mouse hepatitis virus strain A59; MOI, multiplicities of infection; p.i., postinfection.

We first examined the cellular localization of Cx43 in primary murine astrocytes infected with mCoV MHV-A59 at different multiplicities of infection (MOI) 24 h post infection (p.i.). In mock-infected astrocytes, Cx43 localized to puncta at the cell surface consistent with being assembled into gap junction plaques (Fig. 1*A*; small arrows in inset). Upon infection at increasing multiplicities of infection (MOI 2, 5, and 10), Cx43 was retained mainly in the intracellular perinuclear region of cells (Fig. 1, *B*–*D*; large arrows in insets), corroborating our previous findings (31). Viral nucleocapsid (N) staining also colocalized with intracellular Cx43 staining (Fig. 1, *B*–*D*; merged).

Since we previously established that ERp29 is a chaperone that regulates the trafficking and assembly of Cx43 (30), we analyzed ERp29 mRNA and protein expression in mouse primary astrocytes infected with MHV-A59 24 h p.i. There was a significant reduction of ERp29 mRNA expression observed at all MOIs examined (Fig. 1*E*) that was accompanied with a significant decrease in ERp29 protein levels at MOI 5 and MOI 10 (Fig. 1, *F* and *G*). To determine if MHV-induced downregulation of ERp29 was associated with a general ER stress response, the steady-state expression of a classical ER stress marker, BiP/glucose-regulated protein 78, was analyzed (33). At 24 h p.i., BiP mRNA expression was significantly increased at all the MOIs examined (Fig. 1*H*); however, BiP protein levels were only significantly increased at MOI 5 and 10 (Fig. 1, *I* and *J*). The ATF6 pathway, which is one of the major arms of the UPR pathway, also was examined. We found that p90ATF6 was significantly upregulated at MOI 2, MOI 5 and MOI 10 upon MHV-A59 infection for 24 h in mouse primary astrocytes compared to mock infected astrocytes (Fig. 1, *K* and *L*).These data demonstrate that there was a decrease in ERp29 concomitant with increased ER stress in response to MHV-A59 infection.

**Figure 2:**
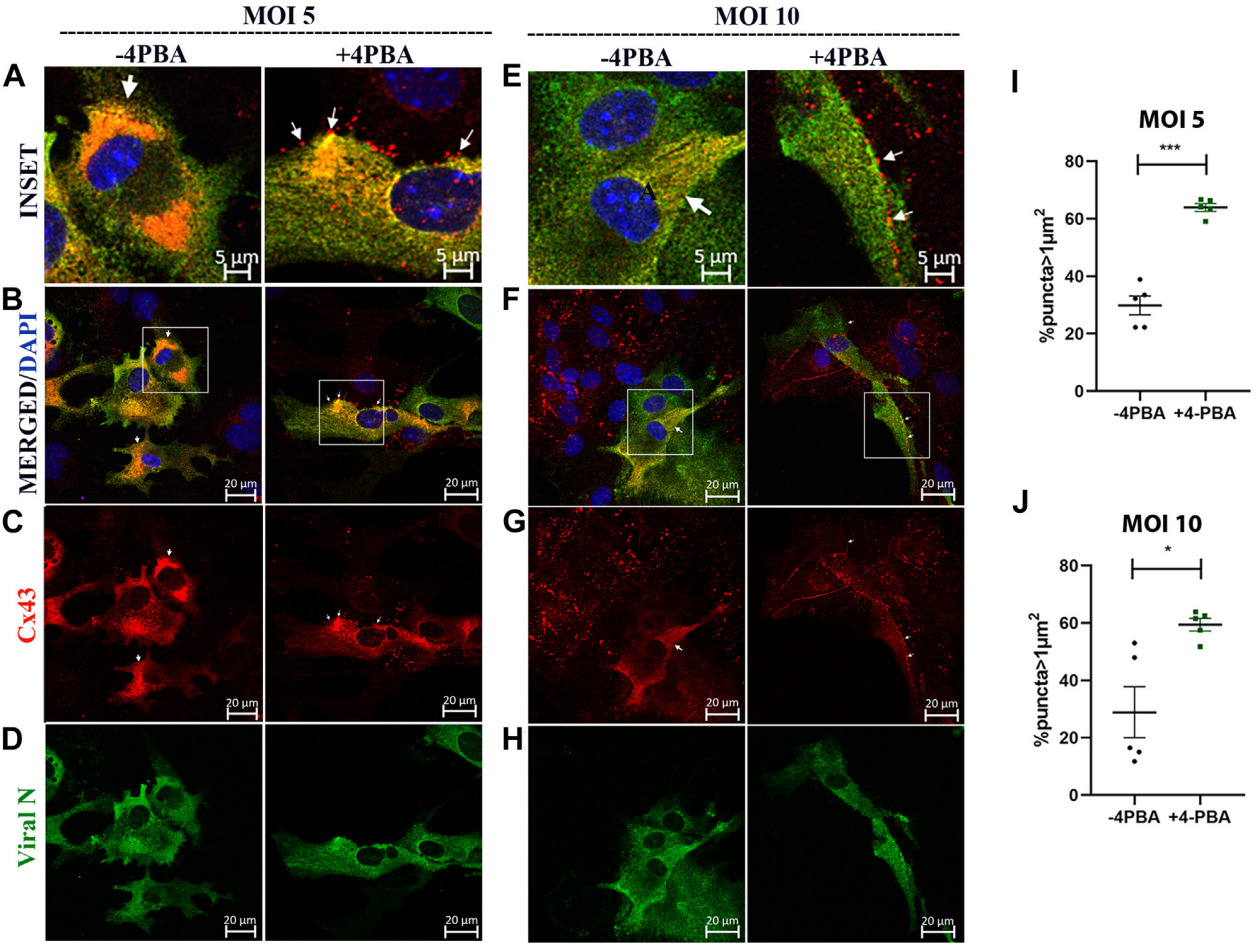
**4-PBA rescues Cx43 trafficking to the cell surface in MHV-A59–infected primary astrocytes**. *A–H*, representative confocal photomicrographs of primary murine astrocytes infected with MHV-A59 at MOI 5 (*A–D*) or MOI 10 (*E–H*) in either the presence or absence of 4-PBA for 24 h. The cells were fixed, immunolabeled for Cx43 (*red*) and viral N protein (*green*) and counterstained with DAPI (*blue*). *Arrows* indicate Cx43 localization in the intracellular compartment in absence of 4-PBA (*A*–*H*: *left panel* and inset) and surface localization of Cx43 in presence of 4-PBA (*A*–*H*: *right panel* and inset). *I* and *J*, scatter plots showing quantification of punctate areas in infected astrocytes with and without 4-PBA. Data from five independent experiments were used for quantification. Values are mean ± SEM, analyzed by unpaired Student’s *t* test (∗*p* < 0.05, ∗∗∗*p* < 0.001, N = 5). 4-PBA, 4-phenylbutyrate; MHV-A59, mouse hepatitis virus strain A59; MOI, multiplicities of infection.

It has previously been demonstrated that the chemical chaperone 4-PBA has the capacity to promote Cx43 assembly into gap junctions as well as upregulate ERp29 (30, 34). To determine if trafficking of Cx43 to the plasma membrane in MHV-A59 infected primary astrocytes can be rescued by 4-PBA, astrocytes infected at MOI 5 and MOI 10 were treated with 10 mM 4-PBA for 24 h, and subsequently the cellular distribution of Cx43 was visualized by immunostaining (Fig. 2, *A*–*H*). In the absence of 4-PBA, MHV-A59 infected astrocytes exhibited intracellular retention of Cx43 (Fig. 2, *A*–*H*, inset, thick arrows). However, 4-PBA treatment of MHV-A59 infected astrocytes restored the ability of Cx43 to form puncta at the cell surface (Fig. 2, *A*–*H*, inset, thin arrows) indicating improved trafficking of Cx43. This was quantifiable, since 4-PBA treatment increased the percentage of Cx43 puncta (plaques) in infected cultures (Fig. 2, *I* and *J*).

**Figure 3:**
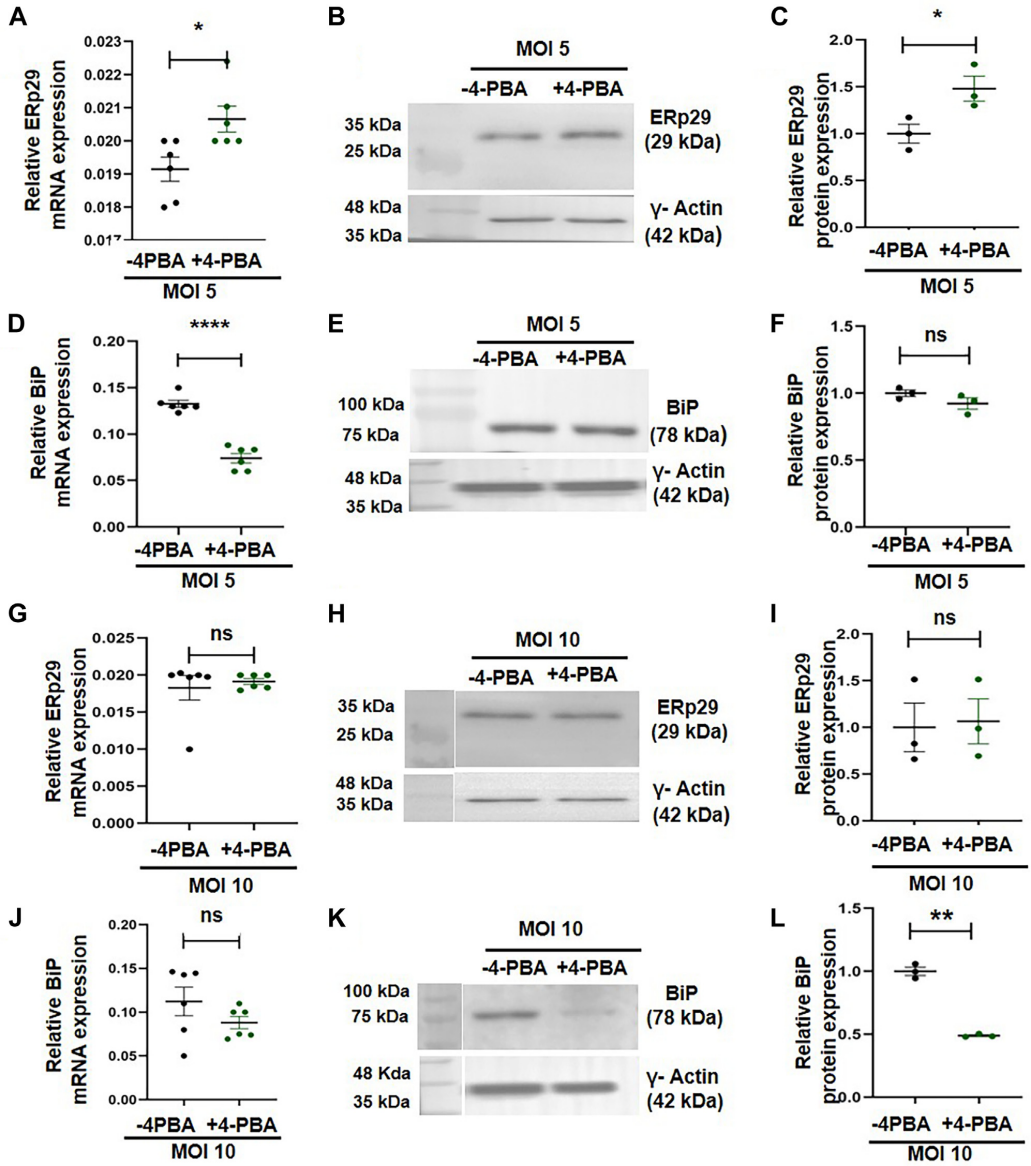
**4-PBA upregulates ERp29 and reduces BiP expression in primary astrocytes infected with MHV-A59.***A*, scatter plot of ERp29 mRNA in MHV-A59–infected astrocytes (MOI 5) in the absence or presence of 4-PBA as measured by qPCR. *B* and *C*, representative immunoblot and corresponding scatter plot of ERp29 protein in MHV-A59–infected astrocytes (MOI 5) in the absence or presence of 4-PBA. γ-Actin was used as a loading control. *D*, scatter plot of BiP mRNA in MHV-A59–infected astrocytes (MOI 5) in the absence or presence of 4-PBA as measured by qPCR. *E* and *F*, representative immunoblot and corresponding scatter plot of BiP protein in MHV-A59–infected astrocytes (MOI 5) in the absence or presence of 4-PBA. *G*, scatter plot of ERp29 mRNA in MHV-A59–infected astrocytes (MOI 10) in the absence or presence of 4-PBA as measured by qPCR. *H* and *I*, representative immunoblot and corresponding scatter plot of ERp29 protein in MHV-A59–infected astrocytes (MOI 10) in the absence or presence of 4-PBA. γ-Actin was used as a loading control. *J*, scatter plot of BiP mRNA in MHV-A59–infected astrocytes (MOI 10) in the absence or presence of 4-PBA as measured by qPCR. *K* and *L*, representative immunoblot and corresponding scatter plot of BiP protein in MHV-A59–infected astrocytes (MOI 10) in the absence or presence of 4-PBA. Values are mean ± SEM analyzed by unpaired Student’s *t* test (∗ *p* < 0.05, ∗∗*p* < 0.01, ∗∗∗∗*p* < 0.0001, ns – not significant, N = 3). 4-PBA, 4-phenylbutyrate; BiP, binding immunoglobulin protein; MHV-A59, mouse hepatitis virus strain A59; MOI, multiplicities of infection.

To determine whether the ability of 4-PBA to rescue Cx43 trafficking in infected cells was due to its impact on virus-altered ERp29 and/or ER stress, we measured ERp29 and BiP in infected cultures (MOI 5), 24 h after 4-PBA treatment at both the mRNA and protein level. Interestingly, ERp29 increased significantly both at RNA and protein levels (Fig. 3, *A*–*C*), while BiP mRNA decreased upon 4-PBA treatment (Fig. 3, *D*–*F*). The ability of 4-PBA to rescue ERp29 expression was saturable, since at MOI 10, there was little effect on ERp29 mRNA or protein expression (Fig. 3, *G*–*I*). However, cells infected at MOI 10 showed significant decreases in BiP expression in response to 4-PBA treatment (Fig. 3, *J–L*), consistent with an ERp29-independent effect of 4-PBA on cell stress.

### Cx43 trafficking and function are impaired in mouse astrocytoma-derived DBT cells and can be rescued by 4-PBA

Further investigating the roles of ERp29 in Cx43–mediated GJIC during acute mCoV infection required a cell line that mimics the effects of MHV-A59 infection on Cx43 expression in primary astrocytes. The mouse astrocytoma-derived DBT cell line (35) provides a homogenous and readily available cell model which was chosen for further investigation. Cultured DBT cells expressed GFAP, confirming their astrocytic nature (Fig. S1*A*). However, unlike primary astrocytes, DBT cells showed significant intracellular retention of Cx43 (Fig. S1*B*), a phenotype that mimics MHV-A59–infected primary astrocytes (Fig. 1). By immunoblot, intracellular retention of Cx43 by DBT cells was accompanied by an altered banding pattern as compared with primary astrocytes (Fig. S1*C*), suggesting differential phosphorylation that correlates with differential Cx43 trafficking (36). Consistent with decreased gap junction channel formation by Cx43, DBT cells also exhibited significantly impaired GJIC-mediated transfer of Lucifer yellow (LY) (Fig. S1, *D* and *E*).

**Figure 4:**
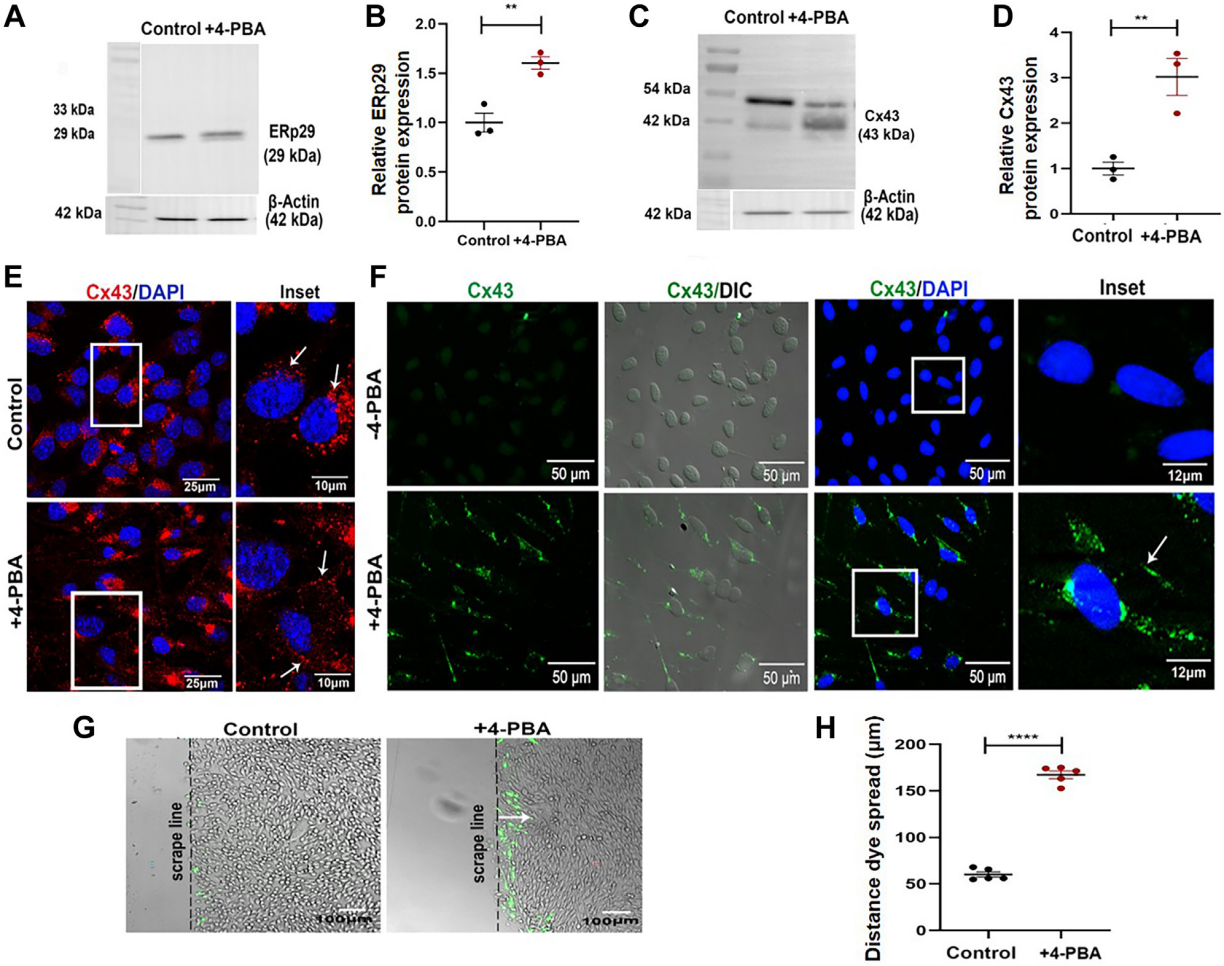
**4-PBA increases ERp29 and Cx43 GJIC in DBT-WT cells**. *A* and *B*, immunoblot and respective scatter plot showing significant upregulation of ERp29 protein level (relative intensity) in control and 4-PBA-treated DBT-WT cells. *C* and *D*, immunoblot and respective scatter plot showing significant upregulation of Cx43 protein in control and 4-PBA-treated DBT-WT cells. *E*, representative images of control and 4-PBA-treated DBT-WT cells immunolabeled with anti-Cx43 (*red*) and counterstained DAPI, showing cellular localization of Cx43 (*arrows*). *F*, fluorescence photomicrographs showing Cx43 gap junction assembly in control (*upper panels*) and 4-PBA-treated DBT-WT cells (*lower panels*) following *in-situ* Triton X-100 solubilization and immunolabeling with anti-Cx43 (*green*). *Arrow* shows Triton X-100 insoluble Cx43 gap junction plaques. *G* and *H*, photomicrographs and scatter plot showing a significant increase in functional GJIC in 4-PBA-treated DBT cells compared to untreated controls. *Arrow* indicates the direction and extent of Lucifer yellow dye transfer (*green*). Values are mean ± SEM analyzed by unpaired student’s *t* test (∗∗*p* < 0.01, ∗∗∗∗*p* < 0.0001, N = 3 or 5). 4-PBA, 4-phenylbutyrate; DAPI, 4, 6-diamidino-2-phenylindole, DBT, delayed brain tumor; GJIC, gap junctional intercellular communication.

To examine if ERp29 can be pharmacologically targeted in DBT cells with 4-PBA, we measured steady-state protein levels of ERp29 in control and 4-PBA–treated cells by immunoblot (Fig. 4, *A* and *B*). ERp29 expression was significantly increased in 4-PBA treated DBT cells as compared to control untreated cells. Treatment with 10 mM 4-PBA for 36 h resulted in a significant increase in the 43 kDa isoform of Cx43 and a corresponding decrease in the 54 kDa band (Fig. 4, *C* and *D*). Overall, total protein levels of Cx43 were significantly increased in 4-PBA–treated DBT cells as compared to control untreated cells.

4-PBA treatment of DBT cells also improved Cx43 trafficking, since 4-PBA–treated DBT cells exhibited distinct punctate staining at the cell surface, indicative of gap junction channel assembly (Fig. 4*E*, arrows in inset). To confirm that Cx43 in 4-PBA–treated DBT cells was assembled into gap junction plaques, we compared control and 4-PBA–treated DBT cells using an *in-situ* Triton X-100 detergent solubilization assay that extracts Cx43 that is not assembled into gap junction plaques (37). Triton X-100 solubilization resulted in a marked reduction of Cx43 staining in control DBT cells, indicating that most of the Cx43 in control DBT cells was not assembled into gap junction plaques (Fig. 4*F*). In contrast, 4-PBA–treated DBT cells had Triton X-100–resistant Cx43 labeling at the cell surface (Fig. 4*F*, inset; arrows). Cx43 plaques present in 4-PBA–treated DBT cells formed functional gap junction channels, as determined using by measuring LY dye transfer by a scrape loading assay (Fig. 4, *G* and *H*).

### MHV-A59 infection of DBT cells results in increased ER stress and reduced ERp29 protein expression

**Figure 5:**
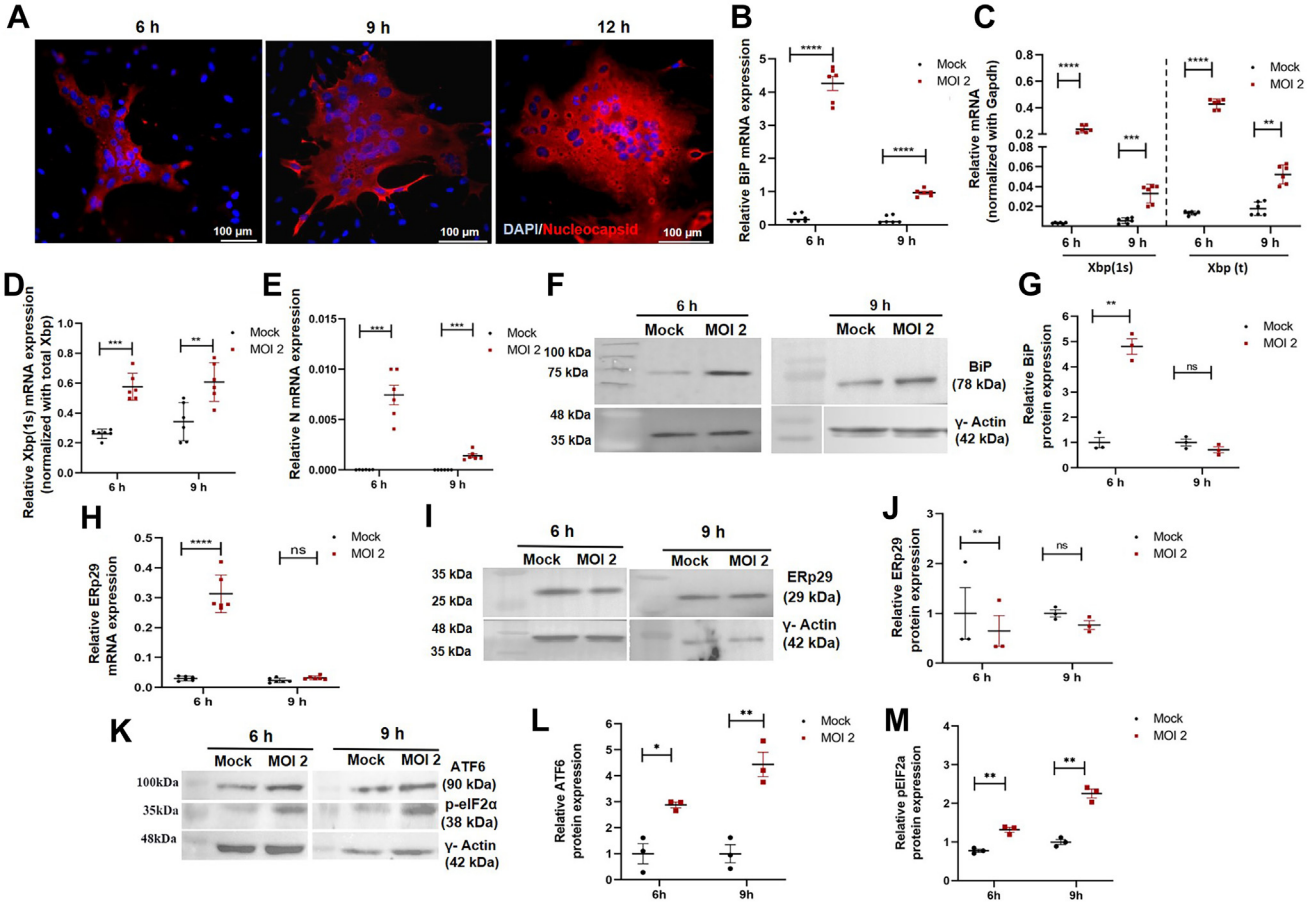
**MHV-A59–induced ER stress and downregulated ERp29 in DBT-WT cells**. *A*, representative microscopic image of DBT-WT cells infected with MHV-A59 at MOI 2 for 6, 9, and 12 h, immunostained with antinucleocapsid (N) (*red*) counterstained with DAPI, showing viral infection and profuse syncytia formation with increasing time p.i. *B–E*, scatter plots showing relative mRNA expression of BiP (*B*), both Xbp-total and Xbp-spliced normalized with Gapdh (*C*), Xbp-spliced normalized with Xbp-total (*D*), and viral nucleocapsid (N) (*E*) in MHV-A59 infected DBT-WT cells at 6 and 9 h p.i. compared to mock infected controls. *F* and *G*, representative immunoblot and corresponding scatter plot showing increased BiP protein in MHV-A59 infected DBT cells at 6 and 9 h p.i. compared to mock infected controls. *H*, scatter plot showing relative ERp29 mRNA expression in DBT-WT cells infected with MHV-A59 at 6 h and 9 h p.i. compared to mock infected controls. *I* and *J*, representative immunoblot and scatterplot showing significant downregulation of ERp29 protein level at 6 h p.i. only. *K–M*, representative immunoblot and scatterplot showing significant upregulation of ATF6 and p-eIF2α protein level at both 6 h p.i. and 9 h p.i. γ-Actin was used as a loading control. Values are mean ± SEM analyzed by unpaired Student’s *t* test or one-way ANOVA (∗*p* < 0.05, ∗∗*p* < 0.01, ∗∗∗*p* < 0.001, ∗∗∗∗*p* < 0.0001, ns – not significant, N = 3). ATF6, activating transcription factor 6; BiP, binding immunoglobulin protein DAPI, 4, 6-diamidino-2-phenylindole; DBT, delayed brain tumor; MHV-A59, mouse hepatitis virus strain A59; MOI, multiplicities of infection.

DBT cells were infected with MHV-A59 at MOI 2 and immunostained for viral nucleocapsid (N) protein at 6, 9, and 12 h p.i (Fig. 5*A*). MHV-A59–infected cells started forming syncytia at 6 h which increased at 9 h p.i. until cells started showing cytopathy at 12 h p.i. (Fig. 5*A*). Thus, we choose to restrict our detailed analysis to MOI 2 at 6 h and 9 h p.i. To determine whether infection with MHV-A59 was associated with a general ER stress response in infected DBT cells, we measured the steady-state expression of the ER stress markers, BiP, total Xbp-1 [total Xbp(t) and spliced Xbp(s)], measured by quantifying spliced Xbp-1[ Xbp(s)] with respect to total Xbp-1 mRNA expression, an indication of IRE1 activation (8, 9, 10). At both 6 h and 9 h p.i., BiP, Xbp(t), and Xbp(s) mRNA showed a significant increase as compared to mock-infected control cells at comparable time points (Fig. 5, *B*–*D*). However, BiP and Xbp(t) mRNA expression was more robust at 6 h than at 9 h p.i, coinciding with higher expression of viral nucleocapsid (N) gene at 6 h p.i. (Fig. 5*E*). However, BiP protein levels only were significantly upregulated at 6 h p.i. suggesting the initiation of a toxic effect of MHV-A59 infection at 9 h p.i. (Fig. 5, *F* and *G*). We then measured ERp29 mRNA expression by infected DBT cells and observed a significant upregulation at 6 h p.i., but not at 9h p.i. (Fig. 5*H*). However, ERp29 protein levels at 6 h p.i. did not correspond with mRNA expression and instead were significantly downregulated (Fig. 5, *I* and *J*). Further analysis of the ATF6 and PERK pathways in DBT-WT cells revealed that p90ATF6 protein levels were significantly upregulated 6 h as well as 9 h post MHV-A59 infection (Fig. 5, *K* and *L*). A similar trend was observed in the level of p-eIF2α protein expression which was significantly upregulated at both 6 h and 9 h p.i. (Fig. 5, *K* and *M*).

**Figure 6:**
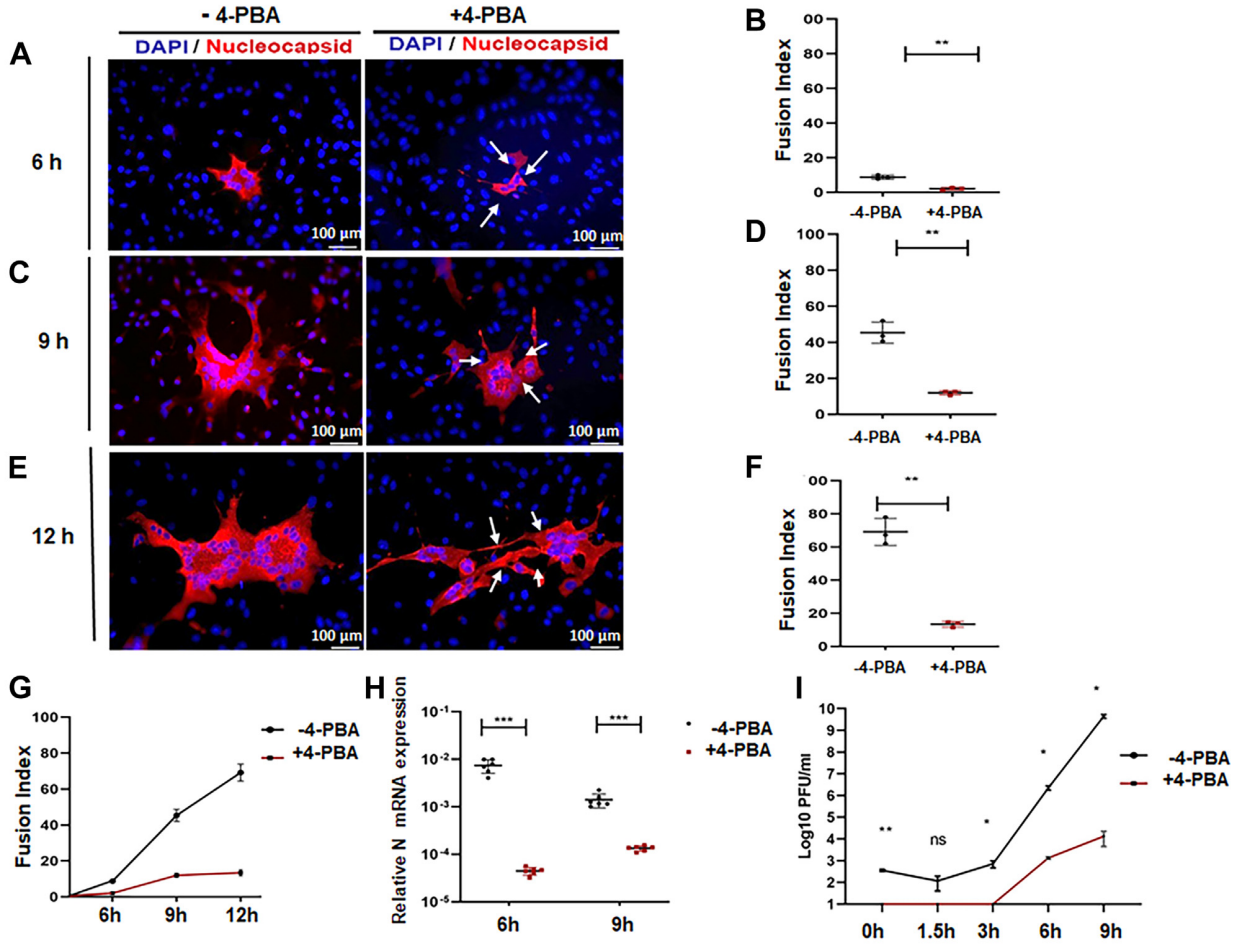
4**-PBA reduces MHV-A59 infectivity in DBT-WT cells**. *A–F*, representative fluorescence photomicrographs and scatter plot showing MHV-A59 induced host cell–cell fusion in DBT-WT cells in the absence or presence of 4-PBA at 6 h (*A* and *B*), 9 h (*C* and *D*), and 12 h p.i. (*E* and *F*). *Arrows* indicate the area of syncytia in control and 4-PBA-treated cells. Scatter plots (*B*, *D*, and *F*) represent a fusion index calculated from 10 fields each from three independent experiments. *G*, kinetics of virus induced cell-cell fusion at 6, 9, and 12 h p.i. in DBT-WT cells with (*red line*) or without (*black line*) 4-PBA. *H*, viral load in MHV-A59–infected DBT-WT cells in the absence or presence of 4-PBA as determined by qPCR for viral N mRNA at 6 h and 9 h p.i. *I*, differential growth kinetics of MHV-A59 in DBT-WT cells with (*red line*) or without (*black line*) 4-PBA treatment determined by calculating viral titer at 0 h, 1.5 h, 3 h, 6 h, and 9 h p.i. Values are mean ± SEM analyzed by unpaired Student’s *t* test or one-way ANOVA (∗*p* < 0.05, ∗∗*p* < 0.01, ∗∗∗*p* < 0.001, ns – not significant, N = 3). 4-PBA, 4-phenylbutyrate; MHV-A59, mouse hepatitis virus strain A59; DAPI, 4, 6-diamidino-2-phenylindole; DBT, delayed brain tumor.

These data suggest that DBT cells were primed for increased toxicity due to MHV-A59 infection, due to deficiencies in regulation of ERp29 and Cx43. Given our demonstration that 4-PBA enhanced Cx43 trafficking (Fig. 2), ERp29 expression (Fig. 3) in MHV-A59–infected primary astrocytes, we examined the effect of 4-PBA on viral spread and infectivity by DBT cells. 4-PBA treatment in MHV-A59–infected DBT cells showed a significant decrease in viral syncytia formation at 6 h, 9 h, and 12 h p.i. compared to untreated infected cultures (Fig. 6, *A*, *C* and *E*; arrows). 4-PBA–treated DBT cells also exhibited delayed kinetics in virus-induced cell–cell fusion compared to untreated infected cells (Fig. 6*G*). Viral N mRNA expression was also significantly downregulated in MHV-A59–infected DBT cells treated with 4-PBA compared to untreated control cultures at 6 h and 9 h p.i. (Fig. 6*H*). The effect of 4-PBA on MHV-A59 replication by DBT-WT cells was measured by performing a standard plaque assay using L2 cells analyzed from 0 to 9 h p.i. (Fig. 6*I*). We found that MHV-A59 showed a slower replication rate in 4-PBA–treated DBT cells than untreated cells. Additionally, 4-PBA also reduced the MHV-A59 viral titer observed at 6 h and 9 h p.i. (Fig. 6*I*). Thus, 4-PBA significantly reduced viral infectivity, nucleocapsid expression, and infectious virus particle production.

### Exogenous ERp29 expression by DBT cells increases Cx43 assembly and attenuates MHV-A59 infection

**Figure 7:**
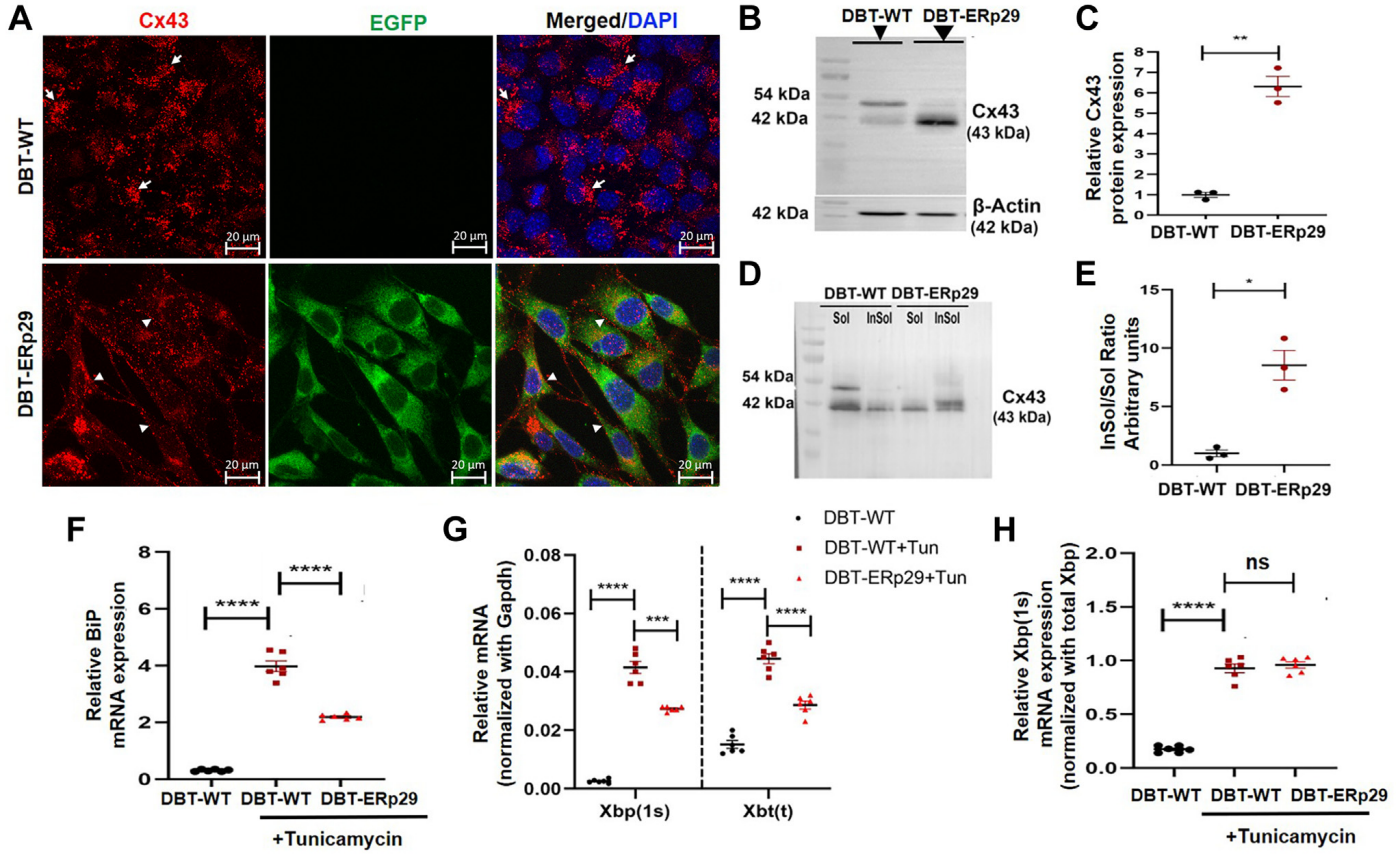
**Exogenous ERp29 expression increases Cx43 trafficking to the cell surface and attenuates ER stress**. *A*, representative images of DBT-ERp29 and DBT-WT cells immunolabeled with anti-Cx43 (*red*) and counterstained with DAPI. *Arrows* indicate intracellular staining of Cx43 in DBT-WT cells (*upper panel*), and *arrowheads* indicate punctate Cx43 staining at cell surface in EGFP-positive DBT-ERp29 cells (*lower panel*). *B* and *C*, immunoblot and respective scatter plot showing significant upregulation of Cx43 protein in DBT-ERp29 and DBT-WT cells. *D* and *E*, immunoblot and respective scatter plot showing Triton X-100 soluble (Sol) and insoluble (InSol) fractions of Cx43. *F–H*, scatter plots showing relative expression of BiP (*F*), Xbp-total, and Xbp-spliced normalized with Gapdh (*G*) and Xbp-spliced normalized with Xbp-total (*H*) mRNA in DBT-WT and DBT-ERp29 cells upon tunicamycin treatment. Tun, tunicamycin. Values are mean ± SEM and analyzed by unpaired Student’s *t* test or one-way ANOVA (∗*p* < 0.05, ∗∗*p* < 0.01, ∗∗∗*p* < 0.001, ∗∗∗∗*p* < 0.0001, ns – not significant, N = 3). DAPI, 4, 6-diamidino-2-phenylindole; DBT, delayed brain tumor.

In order to investigate the specific role of ERp29 in regulating the trafficking of Cx43 and GJIC, we produced DBT cells stably expressing EGFP-tagged ERp29 (referred here as DBT-ERp29) generated by transfection of EGFP-tagged ERp29 into wildtype DBT (DBT-WT), followed by selection (Fig. S2). To measure the effect of increased ERp29 on Cx43, we immunolabeled DBT-ERp29 cells with an anti-Cx43 antibody. In contrast to DBT-WT cells, which showed mostly perinuclear Cx43 (Fig. 7*A*, arrows), DBT-ERp29 cells had prominent punctate Cx43 staining at the cell surface (Fig. 7*A*, arrowheads). The EGFP tag had no effect on Cx43 trafficking, since DBT-WT cells expressing a pEGFP-N1 expression vector showed intracellular retention of Cx43 comparable to DBT-WT cells. Thus, recovery of Cx43 transport to the cell surface in DBT-ERp29 cells required ERp29 expression and was not due to the EGFP tag (Fig. S3).

Additionally, total Cx43 protein levels were increased in DBT-ERp29 cells compared to DBT-WT control cells as determined by immunoblot (Fig. 7, *B* and *C*). The effects of increased ERp29 expression were comparable to that of 4-PBA treatment of DBT-WT cells (Fig. 4), in that DBT-WT cells primarily showed the higher Cx43 band (∼54 kDa) as opposed to DBT-ERp29 cells which predominantly had the 43 kDa band correlating with proper Cx43 trafficking (Fig. 7) (38, 39).

To further examine if DBT-ERp29 cells supported increased assembly of Cx43 into gap junctions, we subjected DBT-WT and DBT-ERp29 cells to the Triton X-100 detergent solubilization assay, using immunoblot as a readout, where Cx43 assembled into gap junctions is resistant to Triton X-100 solubilization. Interestingly, DBT-ERp29 cells showed a significant increase in the ratio of Triton X-100 insoluble to soluble Cx43 levels as compared with DBT-WT cells (Fig. 7, *D* and *E*), further indicating that increased ERp29 expression in DBT cells increased Cx43 gap junction assembly at the cell surface.

To test whether increased ERp29 expression can alter the stress response of DBT cells, we treated cells with chemical ER stress inducer, tunicamycin. Compared with unstressed DBT-WT cells, tunicamycin-treated DBT-WT cells showed an increase in BiP mRNA, Xbp(t) mRNA, and Xbp splicing (Fig. 7, *F*–*H*). By contrast, DBT-ERp29 cells were partially resistant to the effects of tunicamycin, showing less BiP and Xbp(t) mRNA, although Xbp splicing was equivalent for both DBT-WT and DBT-ERp29 cells treated with tunicamycin (Fig. 7*H*).

**Figure 8:**
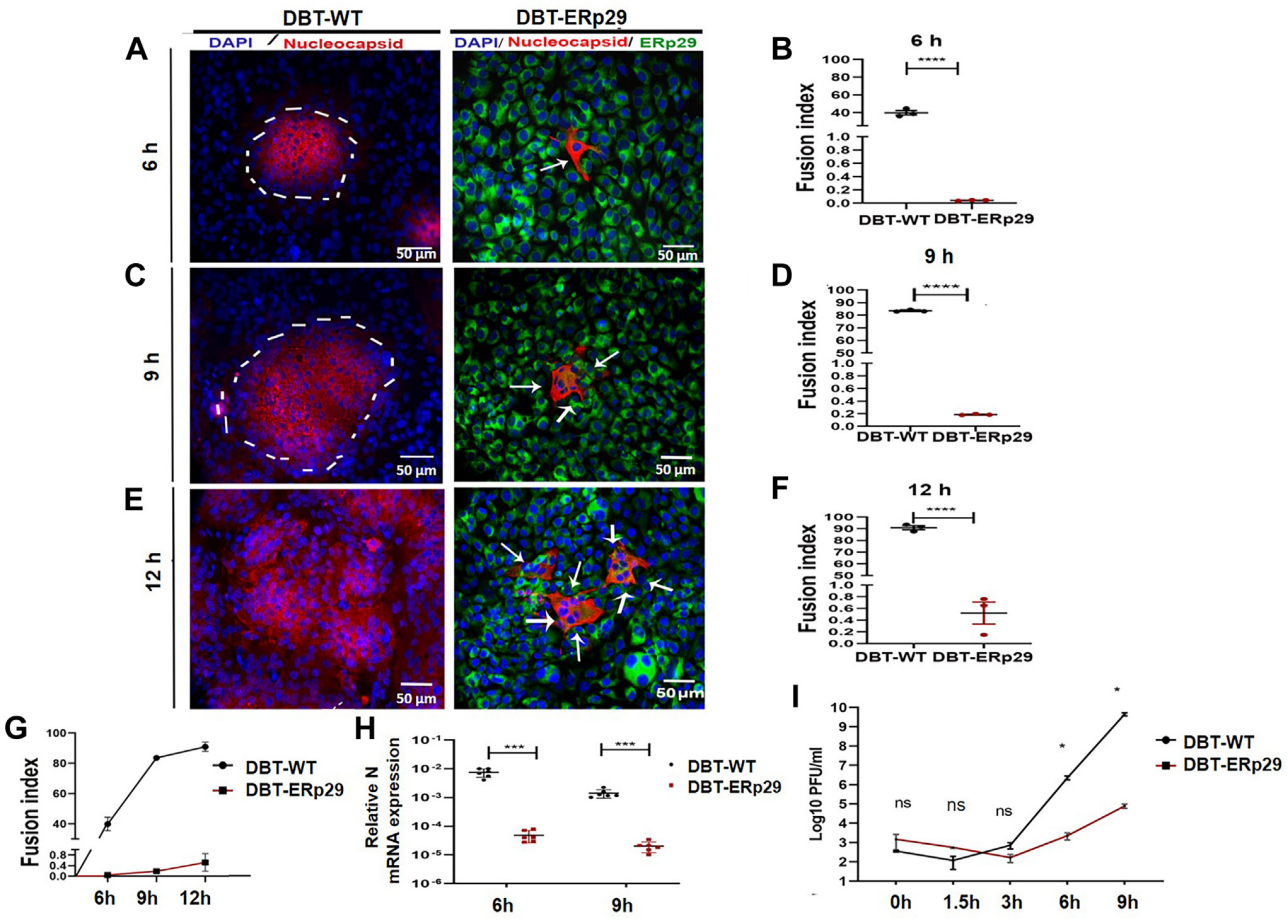
**Exogenous ERp29 expression reduces MHV-A59 infectivity and replication in DBT cells.***A*–*F*, representative confocal images and scatter plots showing host cell–cell fusion in DBT-WT and DBT-ERp29 cells following MHV-A59 infection at 6 h (*A* and *B*), 9 h (*C* and *D*), and 12 h (*E* and *F*) p.i. The perimeter of viral syncytia is indicated by the *dashed area*, *arrows* show restricted syncytia formation by DBT-ERp29. Scatter plots (*B*, *D*, and *F*) represent fusion index calculated from 10 fields, each from three independent experiments. *G*, plot represents kinetics of virus induced cell–cell fusion across 6, 9, and 12 h p.i. in DBT-WT *versus* DBT-ERp29 cells. *H*, viral load in MHV-A59–infected DBT-WT and DBT-ERp29 cells was examined by qPCR for viral N mRNA levels at 6 h and 9 h p.i. *I*, differential growth kinetics of MHV-A59 in DBT-WT (*black line*) *versus* DBT-ERp29 cells (*red line*), estimated by calculating viral titer at 0 h, 1.5 h, 3 h, 6 h, and 9 h p.i. Values are mean ± SEM analyzed by unpaired Student’s *t* test or one-way ANOVA (∗*p* < 0.05, ∗∗∗*p* < 0.001, ∗∗∗∗*p* < 0.001, ns – not significant N = 3). DAPI, 4, 6-diamidino-2-phenylindole; DBT, delayed brain tumor; MHV-A59, mouse hepatitis virus strain A59.

We then determined if increased ERp29 expression in DBT cells affects mCoV infectivity, by analyzing the size, rate, and degree of syncytia formation induced by host cell–cell fusion as a result of MHV-A59 infection (Fig. 8, *A*–*F*). Upon MHV-A59 infection at MOI 2, as shown earlier, DBT-WT control cells formed profuse syncytia in a time-dependent manner. By contrast, DBT-ERp29 cells showed only single-cell infections at 6 h p.i. and significantly restricted syncytia formation at 9 h and 12 h p.i. (Fig. 8, *A*, *C*, and *E*), clearly indicating that increased ERp29 attenuated MHV-A59 infection of DBT cells. This was quantifiable, where DBT-ERp29 cells exhibited delayed virus-induced cell–cell fusion compared to DBT-WT cells (Fig. 8*G*). Also, viral N mRNA expression of MHV-A59 was significantly reduced in DBT-ERp29 cells compared to DBT-WT cells at 6 h and 9 h p.i. (Fig. 8*H*). The replication of MHV-A59 in DBT-ERp29 was compared with that in DBT-WT by analyzing viral growth kinetics from 0 to 9 h p.i. by performing a standard plaque assay in L2 cells. Consistent with our results obtained with 4-PBA–treated DBT-WT cells, MHV-A59 showed a slower replication rate in DBT-ERp29 cells, as evidenced by a lower titer throughout the time course as compared with DBT-WT cells. Furthermore, the MHV-A59 viral titer generated from infected DBT-ERp29 cells was significantly less than that from infected DBT-WT cells at 6 h and 9 h p.i. (Fig. 8*I*). DBT-WT cells transfected with pEGFP-N1 as a negative control showed syncytia formation and infection comparable to untransfected DBT-WT (Fig. S4). DBT-ERp29 cells thus showed a substantial reduction in infectivity, nucleocapsid expression, and replication kinetics of infectious virus particles.

**Figure 9:**
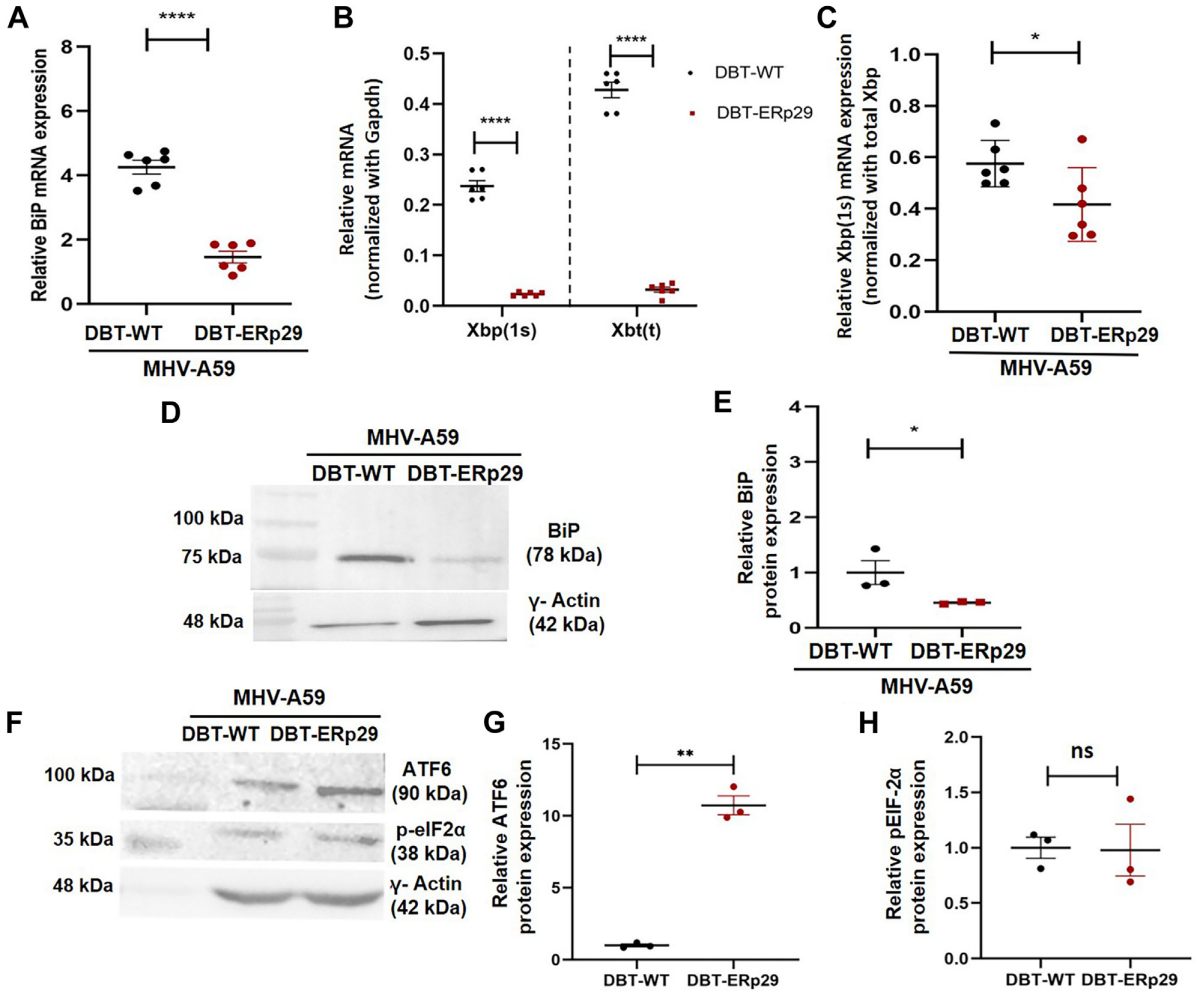
**Exogenous ERp29 expression show reduced ER stress in response to MHV-A59 infection.***A–C*, scatter plot showing relative expression of BiP (*A*), Xbp-total and Xbp-spliced normalized with Gapdh (*B*), and Xbp-spliced normalized with Xbp-total (*C*) in DBT-WT and DBT-ERp29 cells infected with MHV-A59 at 6 h p.i. BiP, Xbp(t) and Xbp(s) mRNA levels show significant downregulation in DBT-ERp29 cells with respect to DBT-WT cells upon MHV-A59 infection. *D* and *E*, immunoblot and respective scatter plot of BiP protein in MHV-A59 infected DBT-ERp29 cells compared to DBT-WT cells. *F*, *G*, and *H*, immunoblot and respective scatter plot of ATF6 and p-eIF2α protein in MHV-A59–infected DBT-ERp29 cells compared to DBT-WT cells. γ-Actin was used as a loading control. Values are mean ± SEM analyzed by unpaired Student’s *t* test (∗*p* < 0.05, ∗∗*p* < 0.01, ∗∗∗∗*p* < 0.0001, N = 3). ATF6, activating transcription factor 6; BiP, binding immunoglobulin protein; DBT, delayed brain tumor; MHV-A59, mouse hepatitis virus strain A59.

We also examined the effect of increased ERp29 on BiP and Xbp in MHV-A59–infected DBT cells at 6 h p.i. when ER stress is at its peak. In comparison to DBT-WT cells, DBT-ERp29 cells showed significantly less BiP mRNA, Xbp(t) mRNA, and Xbp splicing (Fig. 9, *A*–*C*). BiP protein was also significantly lower in infected DBT-ERp29 cells as compared with infected DBT-WT cells (Fig. 9, *D* and *E*). Thus, these results demonstrate a role for ERp29 in specifically preventing replication and spreading of mCoV infection, that may be mediated through modulation of ER stress.

The BiP pathway of UPR was downregulated in MHV-A59–infected DBT-ERp29 compared to DBT-WT. However, analysis of the other two UPR pathways (*i.e*., PERK and ATF6) revealed that p-eIF2α showed no significant differences between the infected DBT-ERp29 and infected DBT-WT cells (Fig. 9, *F* and *H*), while p90ATF6 protein levels were significantly upregulated in infected DBT-ERp29 cells compared to infected DBT-WT cells (Fig. 9, *F* and *G*). Thus, overexpression of ERp29 caused differential upregulation or downregulation of different UPR pathway proteins.

**Figure 10:**
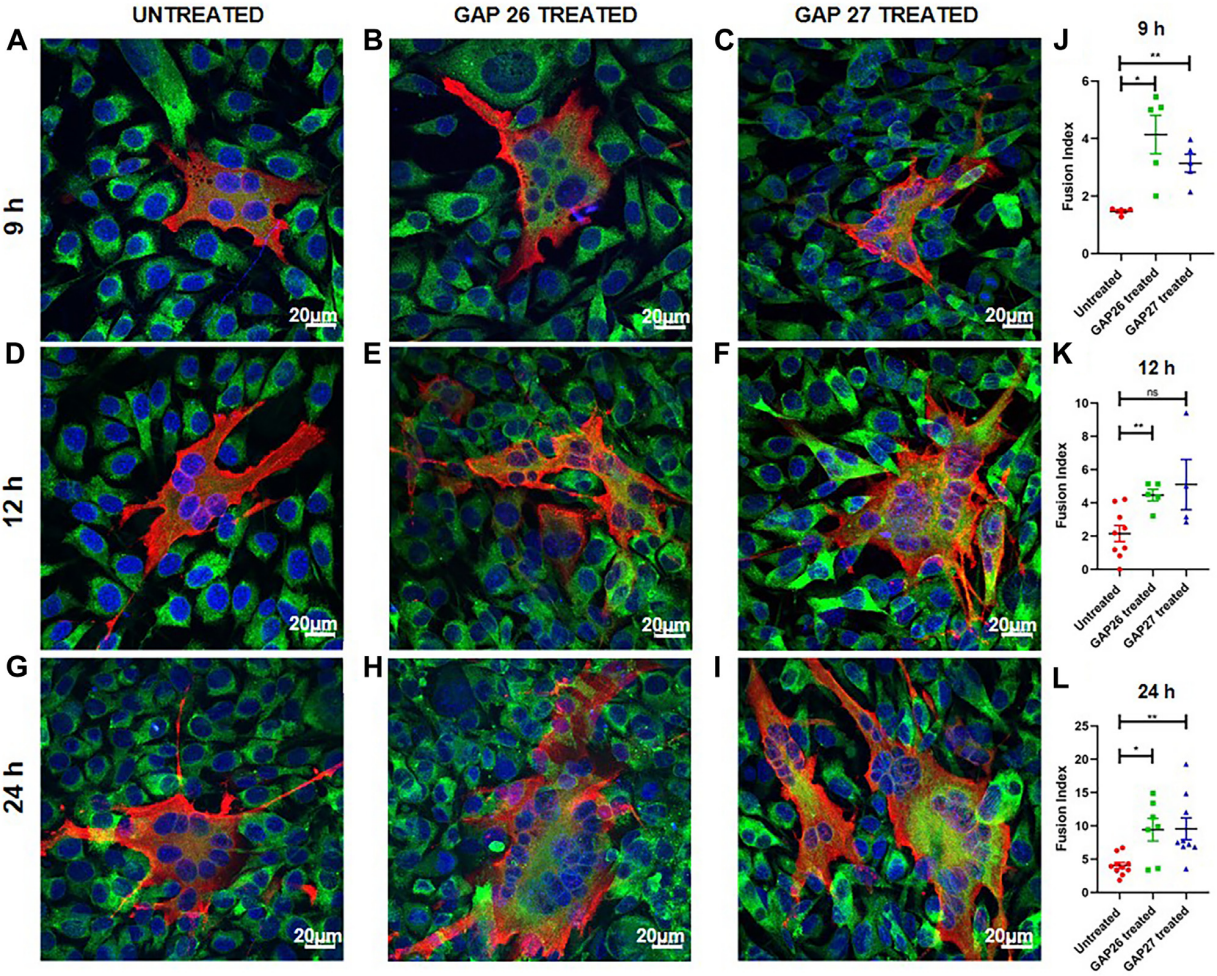
**Inhibition of Cx43 mediated gap junction channels increased viral susceptibility of DBT-ERp29 cells.** Representative confocal images and corresponding scatter plots showing host cell–cell fusion in DBT-ERp29 cells. *A–I*, the cells were either untreated controls (*A*, *D*, and *G*) or treated with Cx43 mimetic peptide GAP 26 (*B*, *E*, and *H*) or GAP 27 (*C*, *F*, and *I*) followed by MHV-A59 infection for 9 h (*A–C*), 12 h (D,E,F), and 24 h (*G–I*) immunolabeled with anti-N (*red*) and counterstained with DAPI in DBT cell line expressing exogenous EGFP-ERp29 (*green*). *J–L*, scatter plots (*J–L*) represent fusion index at 9 h (*J*), 12 H (*K*), and 24 h (*L*), respectively, calculated from 10 fields, each from three independent experiments. Values are mean ± SEM analyzed by unpaired Student’s *t* test (∗*p* < 0.05, ∗∗*p* < 0.01, ns – not significant, N = 3). DBT, delayed brain tumor.

To further determine if the reduced viral infectivity in DBT-ERp29 cells was due to improved Cx43 trafficking to the cell surface and intercellular communication, we examined the effect on infection of two different gap junction peptide mimetics known to inhibit Cx43-mediated gap junctional communication, GAP 26 and GAP 27 (40). DBT-ERp29 cells were pretreated with gap peptides GAP 26 (Fig. 10, *B*, *E*, and *H*), or GAP 27 (Fig. 10, *C, F*, and *I*) and then infected with MHV-A59 at MOI 2. In contrast to untreated controls (Fig. 10, *A, D*, and *G*), cells treated with either GAP 26 or GAP 27 showed quantitative enhancement of syncytia formation (Fig. 10, *J–L*). These data implicate Cx43–mediated gap junctional communication as a downstream mediator of the effect of ERp29 in reducing viral infectivity observed in the DBT-ERp29.

## Discussion

Neurons and glial cells in the CNS are metabolically coupled to each other, forming an interconnected panglial system. Astrocytes are one of the major cell types known to be important for maintaining homeostasis in the panglial system through GJIC by regulating pH, K^+^, and glutamate as well as other solutes. Astrocytes have been shown to express at least three connexins, with Cx43 and Cx30 being well established, and to a lesser extent, Cx26 (41, 42, 43). Cx43 is the most abundant astrocytic connexin expressed *in vivo* and *in vitro*, and its dysfunction is associated with developmental disorders of CNS and several postnatal brain pathologies (44, 45). Previously, we have found that upon MHV-A59 infection, Cx43 is retained in the ER and ERGIC in mouse primary astrocytes (31). Additionally, it was also demonstrated that the virus hijacked the microtubule network needed to facilitate Cx43 transport to the cell surface, which instead promoted viral spreading (32).

The current study extends our previous work by demonstrating that MHV-A59 infection of mouse primary astrocytes and mouse astrocytoma-derived DBT cells lead to increased ER stress and concomitant downregulation of the ER-resident Cx43 chaperone, ERp29. Treatment with 4-PBA attenuated the deleterious effects of MHV-A59 on mouse primary astrocytes, by upregulating ERp29 and improving trafficking of Cx43 to the cell surface. These results were recapitulated in the DBT astrocytoma cell line and, critically, were validated using DBT cells stably transfected to express increased ERp29 (DBT-ERp29 cells). Moreover, transfecting cells with exogenous ERp29 rescued intracellular trafficking of Cx43 to the cell surface. Moreover, re-establishing GJIC was required for reducing susceptibility to viral infection, since mCoV infectivity was significantly increased when Cx43 channels were blocked using connexin-specific mimetic inhibitor peptides in DBT-ERp29 cells. Thus, the current study provides insights into the molecular mechanisms underlying altered trafficking of Cx43 to the cell surface following mCoV infection of astrocytes. Overall, this study establishes novel antiviral roles for ERp29 and Cx43 in restricting viral spread, suggesting their potential utility as pharmacological targets to minimize the deleterious effects of viral infection.

The present study showed MHV-A59 infection induces ER stress in both primary astrocytes and DBT cells, but significantly reduced ERp29 mRNA and protein expression. Conversely, transfecting cells to increase exogenous ERp29 expression significantly reduced mCoV–induced ER stress as measured by levels of BiP and Xbp expressed by DBT-ERp29 cells, measured post infection. Many studies have shown that CoVs such as SARS and MHV can induce ER stress and activate the UPR in infected cells, since assembly of viral structural proteins overwhelms quality control pathways present in the ER and Golgi compartments (46). ER stress induces the UPR pathway, which monitors the protein folding in the ER in an attempt to readjust the folding capacity and quality control of the secretory pathway to match synthesis load (47). Here, we have identified ERp29 as a chaperone that is also regulated by the UPR.

Earlier studies have shown that 4-PBA can enhance Cx43 expression and trafficking (48, 49, 50). Importantly, 4-PBA has been shown to improve trafficking of several mutant transmembrane proteins, including the ΔF508 mutant of cystic fibrosis transmembrane regulator, an effect that is mediated through ERp29 (49, 51). Our results clearly show that 4-PBA improved trafficking and function of Cx43 in MHV-A59–infected mouse primary astrocytes and DBT cells, and this occurs by a mechanism involving increased expression of ERp29. Consistent with a role for ERp29 in the effect of 4-PBA on infected cells, increased expression of transfected ERp29 mimicked the ability of 4-PBA to reduce ER stress and improve trafficking of Cx43 to the cell surface in infected DBT-ERp29 cells.

There is a paradigm shift in our understanding of targeting host cell factors to fight CoV infection. A recent study showed that the human CoVs HCoV-229E, MERS-CoV, and SARS-CoV-2 induced the expression of ER stress proteins like BiP and HERPUD1 at the mRNA level but inhibited their protein expression as a result of global host translational shutoff, similar to the regulation of ERp29 we observed here in DBT cells upon MHV-A59 infection. When ER stress proteins are downregulated, the UPR pathways fail to get induced, leading to impaired protein quality control. By contrast, studies have shown that the induction of the UPR pathway using a well-known chemical activator, thapsigargin, caused a potent inhibitory effect on the replication of three human CoVs in four different cell types (52). The study demonstrated that thapsigargin counteracts the CoV-induced downregulation of BiP and HERPUD1 and increases IRE1α levels to overcome the coronavirus-induced block of global protein biosynthesis (52), thus validating the importance of modulating specific host factors to help reduce the burden of CoV infectivity.

Defining roles for ERp29 and other PDI family proteins in controlling enveloped CoV infection requires additional investigation. In this study, increased ERp29 levels showed a significant reduction in viral infectivity, viral nucleocapsid expression, and viral titer levels, thereby suggesting a novel antiviral role for ERp29 in restricting mCoV replication and spread. We found that regardless of ERp29 expression, cells were equally infected and that viral entry was not reduced.

Supporting our findings, a study on examining MHV-A59 and SARS-CoV2–infected murine fibroblasts and human airway cells showed an upregulation of IRE1α, ATF6, and PERK, the three branches of the UPR (53). Pharmacological inhibition of IRE1α using STF-083010, KIRA8, and AEBSF against ATF6 greatly reduced the replication of both MHV and SARS-CoV-2, revealing the importance of this pathway for successful coronavirus replication (53), further validating that targeting host molecules to restrict CoV infectivity may open a new avenue for designing antiviral therapeutic approaches.

To further investigate if this antiviral response of ERp29 was through restoration of Cx43–mediated GJIC, Cx43 gap junction channels were blocked using the specific Cx43 mimetic peptides Gap 26 and Gap 27, which bind to the first and second extracellular loop regions of Cx43, respectively. The mimetic peptide treatment leads to closure of Cx43 hemichannels in minutes and at a later time points disrupts Cx43–mediated gap junction channel formation (54). The present study demonstrates that the antiviral activity of ERp29 is significantly reduced by mimetic peptides that block Cx43–mediated gap junction channels, thus supporting a role for Cx43 mediated GJIC in reduction of viral infectivity mediated by ERp29.

Our study for the first time demonstrates an anti-coronaviral role for ERp29 which occurs in part through Cx43-mediated GJIC. ERp29 and Cx43 are pharmacologically manipulable and offer important targets with the possible capacity to significantly reduce viral infectivity. This is likely to be advantageous in stimulating an effective host cellular response with the ability to be effective against different strains and mutants of CoVs, providing a potential pan anti-β-coronavirus therapeutic approach.

## Experimental procedures

### Reagents

High-capacity cDNA reverse transcription, DyNAmo Color Flash SYBR Green qPCR, DNase I, the bicinchoninic acid (BCA) protein assay, and enhanced chemiluminescence kits were purchased from ThermoFisher Scientific. RNeasy mini and QIAprep Spin miniprep for RNA and plasmid isolation, respectively, were from Qiagen. Primers for quantitative PCR were obtained from Integrated DNA Technologies (IDT). 4-PBA, tunicamycin, protease, and phosphatase inhibitor cocktails were from Sigma-Aldrich. Cell culture reagents including Dulbecco's modified Eagle's medium (DMEM), Hank's Balanced Salt Solution (HBSS), fetal bovine serum (FBS), 0.25% trypsin, penicillin/streptomycin, fungizone, and G418 were from Thermo Fisher Scientific. VectaShield mounting medium with 4, 6-diamidino-2-phenylindole (DAPI) was from Vector Laboratories, Inc. Sources of all the primary antibodies used in the study are listed in Table 1. Sequences of the primers used in this study are listed in Table 2. All secondary antibodies, conjugated with either horseradish peroxidase, FITC, TRITC, and Texas Red were from Jackson ImmunoResearch Laboratories, Inc. Rest of the chemicals were either from Merck, Amresco, SRL, Sigma-Aldrich, or Thermo Fisher Scientific. The mimetic peptides, GAP 26 and GAP 27, were obtained from Tocris.

**Table 1:** Antibodies used in this study

Sl. No.	Primary antibody	Application	Dilution	Source
1	anti-Cx43 (Polyclonal)	IF WB	1:400 1:1200	Sigma-Aldrich®, MO, USA
2	anti-GFAP (Polyclonal)	IF	1:200	Sigma-Aldrich®, MO, USA
3	anti-Cx43 (Monoclonal)	IF	1:200	Sigma-Aldrich®, MO, USA
4	anti-ERp29 (Polyclonal)	IF WB	1:200 1:1000	Invitrogen, MA, USA
5	Anti-BiP (Monoclonal)	WB	1:1000	Santa Cruz Biotechnology, TX, USA
6	anti-N(Monoclonal)	IF/WB	1:50	Kindly provided by Dr Julian Leibowitz (Texas A&M University College of medicine, TX, USA)
7	anti-γ-Actin/(polyclonal)	WB	1:5000	Bio-Bharati Life Science Pvt. Ltd, India
8	anti-β-Actin (monoclonal)	WB	1:5000	Bio-Bharati Life Science Pvt. Ltd, India
9	anti-ATF-6 (monoclonal)	WB	1:1000	Cell Signaling Technology, MA, USA
10	Anti-Phospho-eIF2a(Polyclonal)	WB	1:1000	Invitrogen, MA, USA

**Table 2:** Sequence of primers used for quantitative real time PCR

Sl. No.	Name of target gene	Forward primer	Reverse primer
1	BiP	5′-TGGCCTGGATAAGAGAGAGGGAGAGAAG3′	5′-GTGTTCCATGACCCGCTGATCAAAGTCT-3′
2	ERp29	5′-GGCAGTTAAGGTTGGAGCCATCCAG-3′	5′- TATGCTGGAGGCCTTGATGAACTCGC-3′
3	Gapdh	5′- GCCCCTTCTGCCGATGC-3′	5′-CTTTCCAGAGGGGCCATCC-3′
4	Viral N	5′-GTTGCAAACAGCCAAGCG-3	5′- GGGCGCAAACCTAGT -3′
5	Xbp(t)	5′- AAGAACACGCTTGGGAATGG-3′	5′- ACTCCCCTTGGCCTCCAC-3′
6	Xbp(s)	5′- GAGTCCGCAGCAGGTG-3′	5′-GTGTCAGAGTCCATGGGA-3′

### Ethics statement

All experimental procedures and animal care and use were strictly regulated and reviewed in accordance with good animal ethics approved by the Institutional Animal Care and Use Committee at the Indian Institute of Science Education and Research Kolkata (AUP no. IISERK/IAEC/AP/2019/29.01). Experiments were performed following the guidelines of the Committee for the Purpose of Control and Supervision of Experiments on Animals, India.

### Isolation of primary mixed glial culture and enrichment of astrocytes

Primary mixed glial cell cultures were established from postnatal day 0 to 1 mouse pups, with minor modifications as previously described (31, 55). Briefly, following the removal of meningeal covering, brain tissues were homogenized and subjected to enzymatic digestion by incubating in a rocking water bath at 37 °C for 30 min in HBSS containing 300 μg/ml DNase I and 10 mg/ml trypsin. Dissociated cells were triturated with 0.25% of FBS in HBSS and passed through a 70-μm nylon mesh. Cells were washed in HBSS at 300*g* for 10 min and plated in astrocyte growth medium containing DMEM supplemented with 10% FBS, 1% L-glutamine, and 1% penicillin and streptomycin. After 24 h, media were changed to remove all nonadherent cells, and cells were allowed to grow to confluency with media change every 3 days thereafter. After 9 to 10 days, once the cultures got confluent, addition of fresh growth medium was stopped for 10 days to allow differential adhesion of astrocytes and microglia. Following this, the culture flasks were thoroughly agitated in an orbital incubator shaker (200 rpm for 40 min at 37 °C), followed by a quick shaking to remove the loosely adherent microglial cells by using differential adherent properties of astrocytes and microglia. The remaining adherent monolayers were enriched in astrocytes.

### Mouse astrocytoma cell culture and generation of stably transfected cells expressing exogenous EGFP-ERp29

We used a mouse astrocytoma-derived DBT cell line (35) which provides a favorable cell culture model of MHV-A59 infection. DBT cells were a kind gift from Dr Stanley Perlman (The University of Iowa) and maintained in DMEM supplemented with 10% heat-inactivated FBS and 1% penicillin-streptomycin at 37 °C with 5% CO_2_ (56).

We used an ERp29 (NCBI: NP_006808.1) EGFP fusion protein in pcDNA3 as previously described (30). Empty vector pEGFP-N1 (Addgene) was used as a negative control. These plasmids were transformed into DH5α competent *E. coli* cells and plasmid DNA isolated using commercially available plasmid isolation kits following manufacturer's instructions. The presence of EGFP-ERp29 gene inserts in the amplified plasmid preparations was confirmed by double digestion using restriction enzymes *BamHI* and *XbaI*. At 60 to 70% confluency, electroporation was carried out following published protocol with minor modifications (57). DBT cells were harvested from T-25 flasks and washed with chilled electroporation buffer (137 mM NaCl, 21 mM Hepes, 0.7 mM disodium phosphate, 6 mM glucose; pH 7.4). Equal volumes of DBT cell suspension and 30 μg of EGFP-ERp29 or pEGFP-N1 plasmid DNA were mixed with electroporation buffer, incubated on ice for 10 min, and subjected to a short electric pulse of 1200 V, 10 μF for 3 milliseconds in Gene Pulser Xcell electroporation system (Bio-Rad). Following a brief incubation on ice for 10 min, the cells were seeded in a 100 mm dish and allowed to grow at 37 °C with 5% CO_2_ for 48 h in DMEM. Subsequently, DMEM was replaced with DMEM containing 2 mg/ml G418. Stably transfected individual cells were subjected to colony selection and expansion to generate a DBT cell line expressing exogenous EGFP-ERp29 (referred here as DBT-ERp29) or only EGFP (referred as DBT-EGFP).

### Mouse hepatitis virus infection

A neurotropic demyelinating strain of mouse hepatitis virus, MHV-A59, was used for infecting mouse DBT cells following previously published protocols from our previous studies (31). In brief, cultures grown to confluency were infected with MHV-A59 diluted in inoculation medium (DMEM containing 2% FBS) at MOI of 2 and incubated for 1 h at 37 °C in a humidified CO_2_ chamber. After initial viral adsorption for 1.5 h, the inoculation medium was discarded, and infected cells were maintained in normal growth medium for 6, 9, or 12 h. For mock infection, parallel cultures were initially incubated in the inoculation medium for 1.5 h followed by normal growth medium. All cells were collected at the respective time-points p.i. and used for immunolabeling and biochemical assays as described below.

### 4-PBA and tunicamycin treatment

MHV-A59–infected mouse primary astrocytes were either treated with 10 mM 4-PBA or maintained in normal growth medium without 4-PBA for 24 h. DBT cells were treated with 10 mM 4-PBA for 36 h. Immunofluorescence and immunoblot experiments were carried out in the 4-PBA- and mock-treated cells according to the protocol described below.

ER stress has been shown to be induced by treating cells with 2.5 to 5 μg/ml of tunicamycin for 5 to 6 h (20). For our experiments, cells were treated with normal growth medium supplemented with 2.5 μg/ml tunicamycin for 6 h. RNA isolation and quantitative PCR was performed as described below from control and tunicamycin-treated cells.

### Immunofluorescence microscopy

Cells were allowed to grow to confluency and infected with MHV-A59 or mock-infected and immunostained as described previously (31). Briefly, at 24 h p.i., the cells were fixed with 4% paraformaldehyde for 15 min at room temperature (RT). Subsequently, they were permeabilized with 0.5% Triton X-100 in phosphate buffered saline (PBS) and blocked using 2.5% heat-inactivated goat serum in 1× PBS. Initially, cells were incubated with the respective primary antibodies diluted in the blocking solution for 1 h at RT at varying dilutions (Table 1). Cells were then washed and incubated with FITC, Texas Red diluted, or TRITC-conjugated secondary antibodies (1:200) in blocking solution for 1 h at RT. Finally, the cells were washed in PBS and mounted using Vectashield with DAPI.

Confocal imaging was done using Zeiss Confocal Microscope (LSM710), and epifluorescence images were acquired using a Nikon eclipse Ti2 epifluorescence microscope (TYO) and DS Qi2 device camera (TYO). Images processed with Zen 2012 (Carl Zeiss AG), ImagePro (Media Cybernetics), and ImageJ (NIH) software.

### Immunoblot

Cells were homogenized in protein extraction buffer (25 mM Tris (pH 7.6), 1 mM MgCl_2_, 1% Triton X-100, and 0.5% SDS) comprising EDTA-free complete protease and phosphatase inhibitors (1 mM NaVO_4_ and 10 mM NaF) by vortexing at regular intervals for a period of 1 h. Subsequently, cell lysates were centrifuged at 13,200 rpm for 20 min at 4 °C using an Eppendorf 5415 R centrifuge. Protein concentrations were determined using a Pierce BCA assay kit following manufacturer's instructions. Equal amounts of protein for mock and virus-infected or 4-PBA-treated samples were resolved by 10% or 12% SDS-PAGE gel electrophoresis and transferred to PVDF membranes. Membranes were blocked with 5% skim milk in Tris-buffered saline containing 0.1% Tween-20 for 1 h at RT and subsequently incubated with the primary antibodies diluted in blocking solution as listed in Table 1, overnight at 4 °C. After washing, membranes were incubated with horseradish peroxidase–conjugated secondary antibodies prepared in blocking solution at 1:10,000 dilution for 1 h at RT. Membranes were washed in 1× Tris-buffered saline containing 0.1% Tween-20, and immunoreactive bands were seen using Super Signal West Pico chemiluminescent detection kit following manufacturer's protocol. Images were captured using a Syngene G: Box ChemiDoc system furnished with GENESys software (Genesys). All the blots were re-probed with anti-γ-actin or anti-β-actin antibody to ensure equal protein loading. Densitometric image analysis was done using ImageJ (Image J, National Institutes of Health).

### Quantitative real-time PCR

RNA was isolated from mock and infected mouse primary astrocytes and DBT cells using Qiagen RNeasy minikit according to the manufacturer's instructions. cDNA was synthesized from 1 μg total RNA using high-capacity cDNA reverse transcription kit following manufacturer's protocol. Quantitative real-time PCR was performed using DyNAmo Color Flash SYBR Green master mix in Applied Biosystems 7500 Real-Time PCR System (Applied Biosystems, Thermo Fisher Scientific). Thermal cycling conditions were comprised of initial denaturation at 95 °C for 7 min, 40 cycles of 95 °C for 10 s, and 60 °C for 30 s. Melting occurred at 60 °C for 30 s. Samples were loaded in duplicates for the gene of interest and internal control Gapdh from three independent biological sets, using specific primers as listed in Table 2. A comparative cycle threshold method was employed to calculate the relative mRNA expression (2^-ΔCt^) after normalization with internal control Gapdh.

### Estimation of viral fusogenicity

Monolayers of DBT-WT, DBT-ERp29, and DBT-EGFP cells grown on coverslips were infected with MHV-A59 at MOI 2 for 6 h, 9 h, and 12 h. Also, DBT-WT cells were treated with 4-PBA p.i. with MHV-A59. Cells were immunostained for viral N and subsequently fixed with 4% PFA and mounted on glass slide using Vectashield with DAPI to stain all nuclei. The cells were then visualized using an Olympus IX-81. Images were acquired using an Orca-1 charge-coupled device and camera (Hamamatsu) and Image-Pro image analysis software (Media Cybernetics, Silver 99 Spring). Syncytia formation was quantified for each condition, from three independent biological sets, in terms of fusion index using the following formula: Fusion Index = (N-S)/T × 100, where N denotes the number of nuclei in the syncytia, S indicates the number of syncytia, and T denotes the total number of nuclei (58, 59). Kinetics of virus-induced cell–cell fusion was determined by plotting the fusion index values against the respective times in GraphPad Prism.

### Estimation of viral growth kinetics by titer assay

For viral growth kinetics, DBT-WT and DBT-ERp29 cells were grown in 35-mm culture dishes. Almost 80% confluent monolayers of cells were infected with MHV-A59 at an MOI of 2. After 1.5 h of viral adsorption, the infected cells were washed and placed in fresh medium with or without 4-PBA and collected at 0, 1.5, 3, 6, or 9 h for performing three freeze-thaw cycles at minus 80 °C and RT. Finally, lysed cells were centrifuged at 500 rpm for 15 min at 4 °C. Supernatants were subjected to a routine plaque assay for titer estimation according to the standard plaque assay protocol (58, 60, 61). Briefly, mouse L2 fibroblast cells seeded in 6-well plates grown to confluent monolayers in growth medium containing DMEM supplemented with 10% FBS, 1% Hepes, 12.5 mM sodium bicarbonate, and 1% penicillin–streptomycin. Total virus in the cell supernatant was then serially diluted in DMEM containing 2% FBS. Two hundred microliter from each of the serially diluted viral inoculum was used to infect L2 cell monolayer in each well. The plates were incubated at 37 °C with 5% CO_2_ for 1h with intermittent shaking at every 15 min. Cells were then washed and overlaid with 0.7% agarose (1 volume of 1.4% dissolved agarose in 1× PBS with 1 volume of 2× L2 medium). The agarose medium was allowed to solidify and then incubated at 37 °C with 5% CO_2_ for 20h to allow plaque formation. The layer of agarose was scraped out and cells fixed with 4% PFA and were stained with crystal violet to visualize the infectious plaques. Viral titer was calculated according to the following mathematical formula: log10 (number of plaques X dilution factor/ml).

### Triton X-100 solubilization assay

Cells were harvested in ice-cold 1× PBS containing protease and phosphatase inhibitors and passed through a Dounce homogenizer for 100 times. The homogenate was centrifuged at 500g for 5 min in an Eppendorf 5415 R centrifuge, and supernatant collected was subsequently centrifuged at 100,000*g* for 30 min using Beckman Optima Max ultracentrifuge. The membrane-enriched pellet was resuspended in ice-cold 1× PBS containing 1% Triton X-100 and incubated for 30 min at strictly maintained at 4 °C (62). The sample was then centrifuged at 100,000*g* for 30 min to separate Triton X-100 soluble (supernatant) and insoluble fractions (pellet), respectively. Both the soluble and insoluble fractions were diluted in Laemmli sample buffer, and equal volumes were resolved by SDS-PAGE followed by immunoblot to detect Cx43. For *in situ* Triton X-100 extraction, cells grown on coverslips were rinsed with 1× PBS and treated with 1% Triton X-100 for 30 min at 4 °C. The cells were then washed, fixed with 4% PFA, and immunostained as described previously.

### Dye transfer assay

Functional GJIC was evaluated by LY dye transfer in a scrape loading assay as previously described (63). Confluent monolayers of DBT cells grown on 35 mm dishes were washed with 1× PBS. 1× PBS containing 4 mg/ml LY was scrape loaded in the cell monolayer and incubated for 1 min at RT. Growth media were added following PBS wash, and live imaging was performed to monitor dye transfer to gap junction coupled cells. GIJC was then calculated as the distance of LY transfer from the scrape loading line using ImageJ software.

### Cx43 mimetic peptide treatment

The mechanisms of action for Gap26 and Gap27 mimetic peptides have been extensively studied (64, 65, 66). Gap26 and Gap27 peptides bind to Cx43 extracellular loop domains 1 and 2, respectively, and close Cx43 hemichannels within minutes, while disruption of gap junction–mediated cell coupling occurs at later timepoints (30 min or longer). Hemichannels with bound peptides move laterally toward the rims of gap junction plaques as they assemble and are then inactivated by being internalized (65). Also, the MM/generalized born surface area ΔGbind scores of peptides docked to free Cx43 hemichannels were -100 for Gap26 and -80 for Gap27, indicating high affinity binding (67) DBT-ERp29 cells were pretreated with either 200 μM Gap 26, 300 μM Gap 27 or maintained in normal growth medium without any mimetic peptide for 24 h prior to infection with MHV-A59 which is known to block Cx43–mediated GJIC (54). Immunofluorescence for viral N was carried out subsequently at 9, 12, and 24 h, followed by fusion index calculation according to the formula described above.

### Statistical analysis

Data presented are as mean ± SEM (standard error of the mean). *p* value for the graphical data was generated using either two-tailed Student's *t* test with Welch's correction or One-way analysis of variance (ANOVA) followed by multiple comparison using Tukey's test for correction. Statistical significance was set at *p* < 0.05. All statistical analyses were carried out using GraphPad Prism 8 software (GraphPad Software, Inc).

## Data availability

Cell lines as well as data supporting the study findings can be obtained from the corresponding author on request.

## Supporting information

This article contains supporting information (Figs. S1, S2, S3 and S4).

## Conflict of interest

The authors declare no conflict of interest with the contents of the article.
