# Supplementary material for: Regulatory role of endoplasmic reticulum resident chaperone protein ERp29 in anti-murine β-coronavirus host cell response
Source: J Biol Chem. 2022 Dec 23;299(2):102836. doi: 10.1016/j.jbc.2022.102836 (PMC9788854; doi:10.1016/j.jbc.2022.102836)
Supplement: Supplementary figures [file mmc1.docx]

**Supplementary Figures**


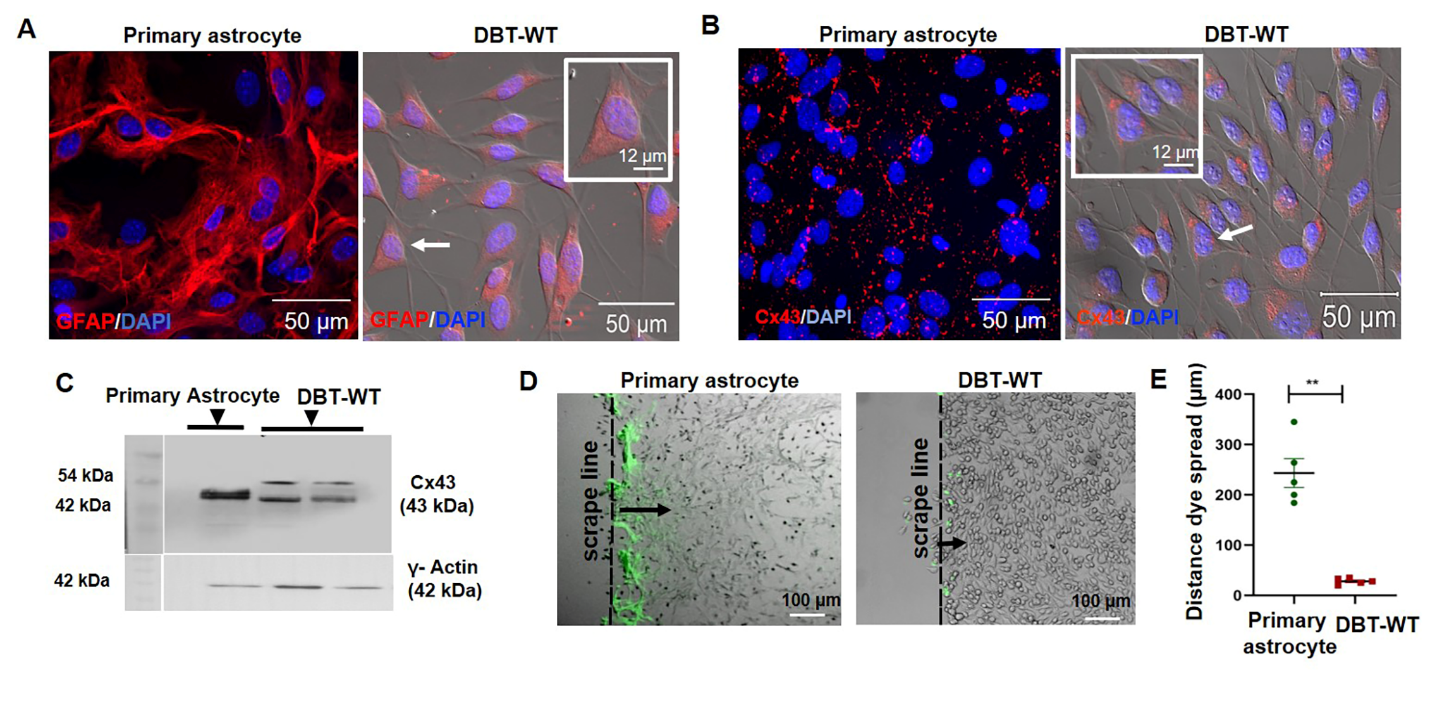


**Figure S1.** **Mouse astrocytoma-derived DBT-WT cells show intracellular Cx43 retention mimicking MHV-A59 infected mouse primary astrocytes.** (A) Representative immunofluorescence images of primary astrocytes and DBT-WT cells immunolabelled with anti-GFAP (red). (B) Representative immunofluorescence images of primary astrocytes and DBT-WT cells immunolabelled with anti-Cx43 (red) showing intracellular retention of Cx43 in DBT-WT cells. DBT-WT cell images are superimposed on DIC images. (C) Immunoblot showing reduced phosphorylation of Cx43 protein in DBT-WT cells compared to primary astrocytes. (D,E) Representative photomicrographs and scatter plot demonstrating GJIC in primary astrocytes and DBT-WT cells. Arrow indicates the direction and distance of Lucifer Yellow dye transfer (green). Values are mean ± SEM analyzed by unpaired Student’s t-test (**p<0.01, N=3 or 5).


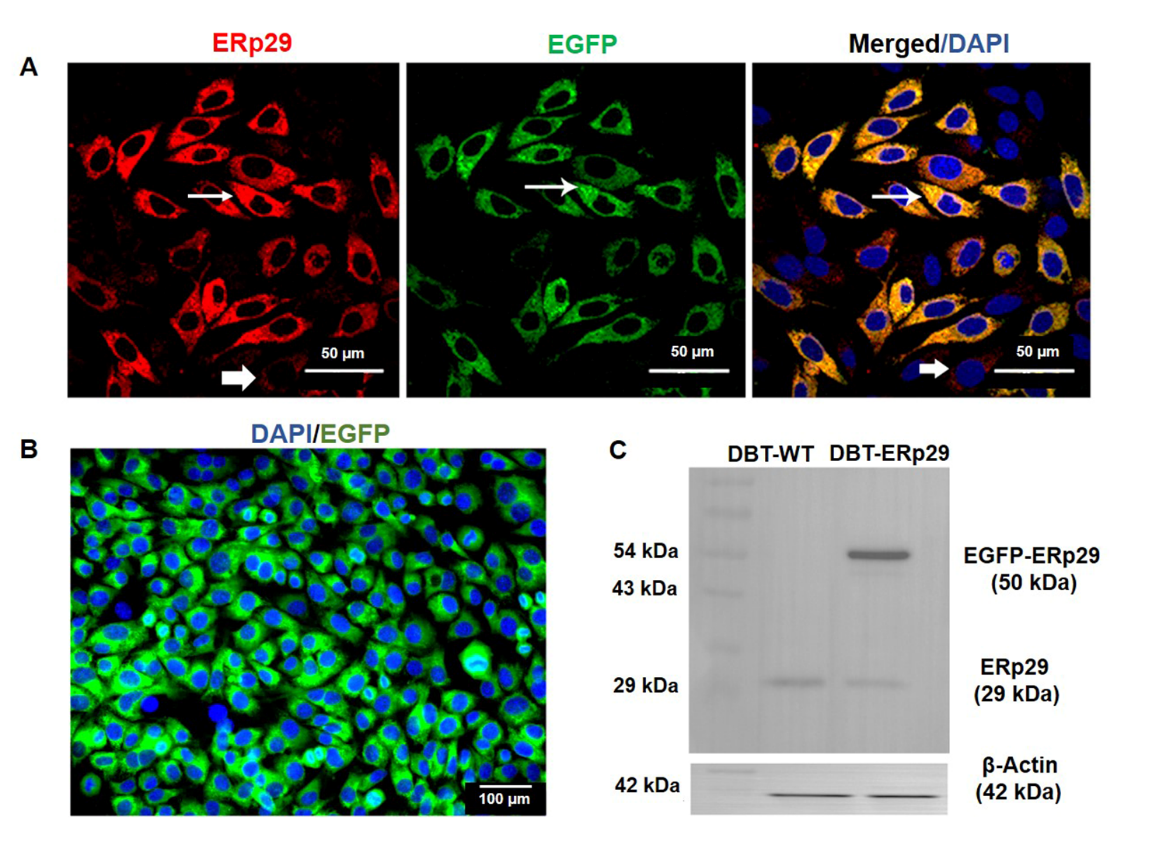


**Figure S2.** **Transfection of exogenous EGFP-ERp29 into DBT cells.** (A) Representative confocal photomicrographs of DBT cells transiently transfected with EGFP-ERp29 show expression of EGFP (green) tagged ERp29 (red) (thin arrow), counterstained with DAPI. Untransfected cells lacking EGFP expression show endogenous ERp29 staining (thick arrow). (B) Following selection by culture in G418-containing medium, stably transfected DBT-ERp29 sells showed EGFP expression (green) in all cells. (C) Immunoblot showing the EGFP-ERp29 fusion protein (50 kDa) in DBT-ERp29 cells compared to endogenous ERp29 (29 kDa).


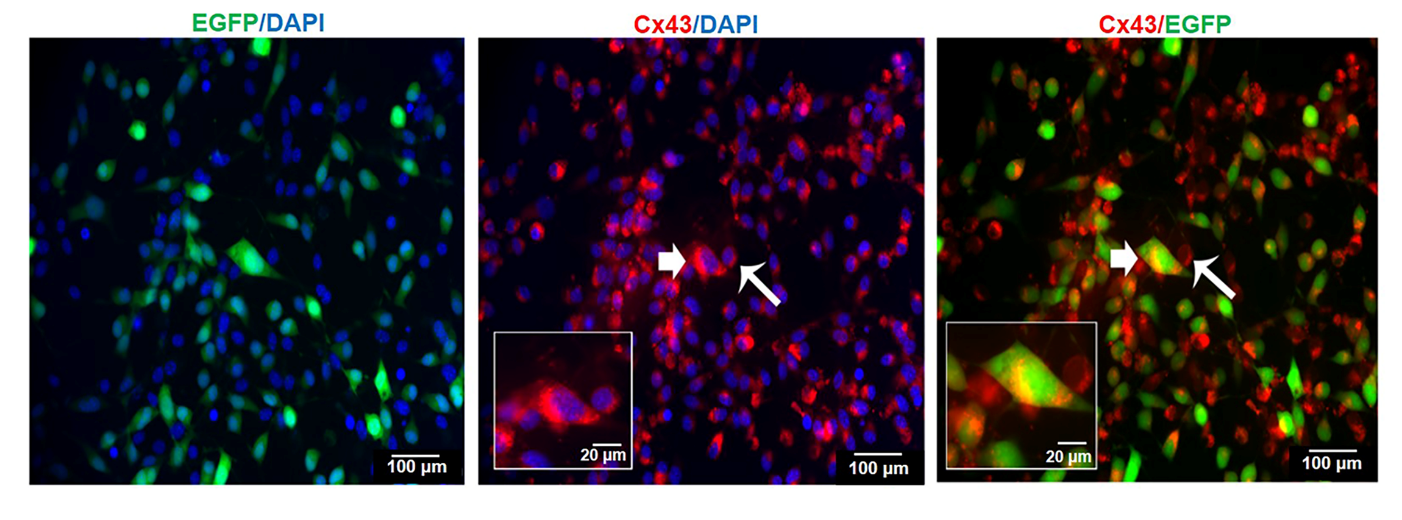


**Figure S3. EGFP alone does not alter Cx43 transport to the cell surface in DBT cells.** Representative micrograph of DBT-EGFP cells immunolabelled with anti-Cx43 (red) and counterstained with DAPI, showing perinuclear Cx43 immunostaining in cells expressing EGFP (green, thick arrow). The thin arrow marks an EGFP-negative DBT-WT cell in the culture also showing perinuclear Cx43.


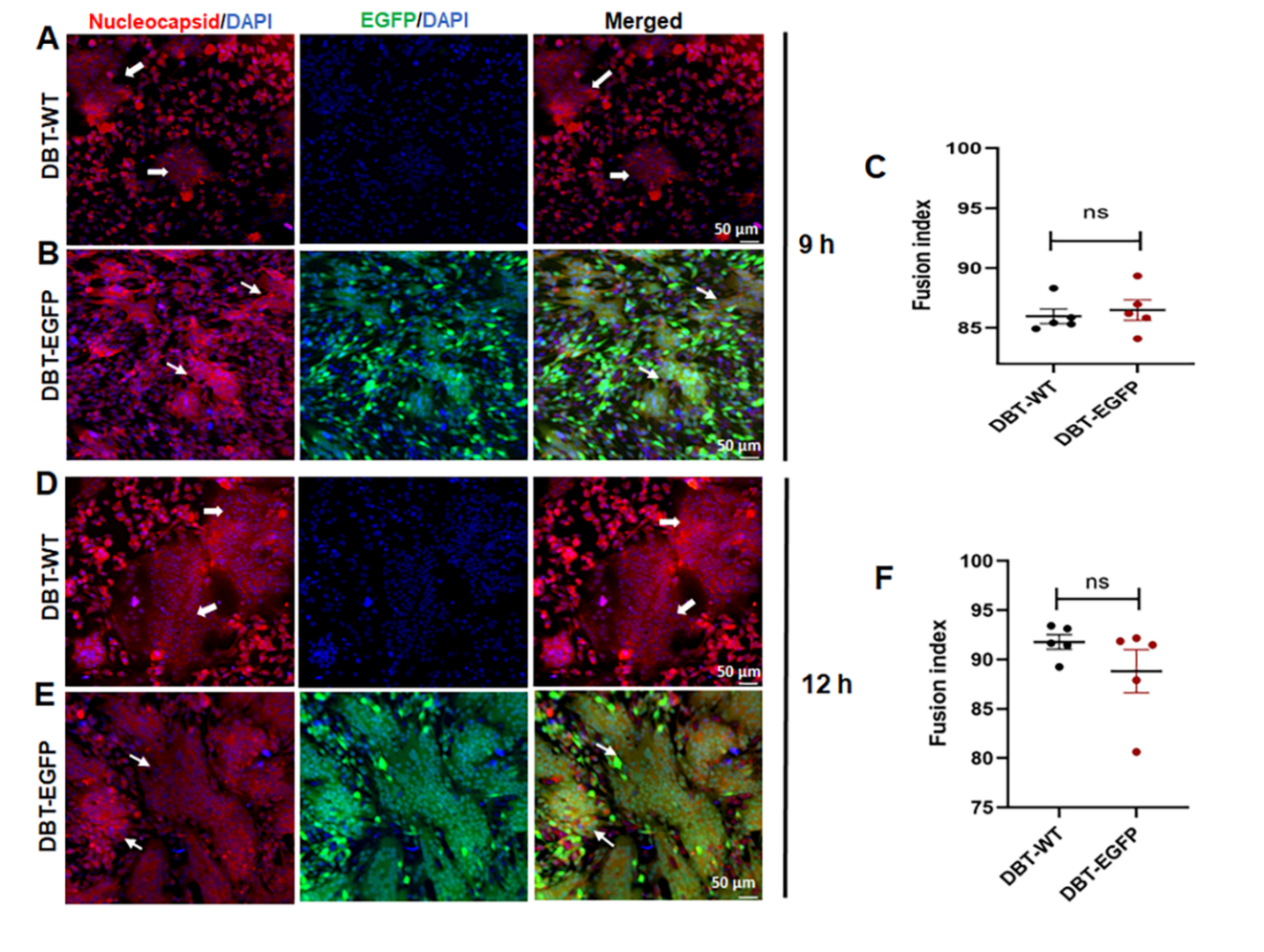


**Figure S4.** **EGFP alone does not alter MHV infectivity and syncytia formation in DBT cells.** (A, B) Representative fluorescence photomicrographs of DBT-WT cells (A) and DBT-EGFP cells (B) infected with MHV-A59 at 9 h p.i. immunolabelled with anti-nucleocapsid (N) (red) and counterstained with DAPI. Cells expressing EGFP (green, thin arrows) show syncytia formation comparable to DBT-WT cells (thick arrows). (C) Scatter plot representing fusion index shows no significant difference in degree of cell-cell fusion between DBT-WT and DBT-EGFP at 9 h p.i. (D, E) Representative fluorescence photomicrographs of DBT-WT cells (D) and DBT-EGFP cells (E) infected with MHV-A59 at 12 h p.i. immunolabelled with anti-nucleocapsid (N) (red) and counterstained with DAPI. Cells expressing EGFP (green, thin arrows) show syncytia formation comparable to DBT-WT cells (thick arrows). (F) Scatter plot representing fusion index shows no significant difference in degree of cell-cell fusion between DBT-WT and DBT-EGFP at 12 h p.i. Fusion index was calculated from 10 fields, each from 5 independent biological experiments. Values are mean ± SEM analyzed by unpaired Student’s t test (ns – not significant, N=5).
